# European Project on Osteoarthritis (EPOSA): methodological challenges in harmonization of existing data from five European population-based cohorts on aging

**DOI:** 10.1186/1471-2474-12-272

**Published:** 2011-11-28

**Authors:** Laura A Schaap, Geeske MEE Peeters, Elaine M Dennison, Sabina Zambon, Thorsten Nikolaus, Mercedes Sanchez-Martinez, Estella Musacchio, Natasja M van Schoor, Dorly JH Deeg

**Affiliations:** 1 Department of Epidemiology and Biostatistics, EMGO Institute for Health and Care Research, VU University Medical Center, Amsterdam, the Netherlands; 2 School of human movement studies, University of Queensland, Brisbane, Australia; 3 MRC Lifecourse Epidemiology Unit, University of Southampton, Southampton General Hospital, Southampton, UK; 4 Department of Medical and Surgical Sciences, University of Padova, and National Research Council (CNR), Aging Branch, Institute of Neuroscience, Padova, Italy; 5 AGAPLESION Bethesda Clinic Ulm, University of Ulm, Ulm, Germany; 6 Department of Preventive Medicine and Public Health, Unit of Primary Care and Family Medicine, Faculty of Medicine, Universidad Autonoma de Madrid, Madrid, Spain

## Abstract

**Background:**

The European Project on OSteoArthritis (EPOSA), here presented for the first time, is a collaborative study involving five European cohort studies on aging. This project focuses on the personal and societal burden and its determinants of osteoarthritis (OA). The aim of the current report is to describe the purpose of the project, the post harmonization of the cross-national data and methodological challenges related to the harmonization process

**Methods:**

The study includes data from cohort studies in five European countries (Germany, Italy, the Netherlands, Spain and the United Kingdom) on older community-dwelling persons aged ≥ 59 years. The study design and main characteristics of the five cohort studies are described. Post harmonization algorithms are developed by finding a "common denominator" to merge the datasets and weights are calculated to adjust for differences in age and sex distribution across the datasets.

**Results:**

A harmonized database was developed, consisting of merged data from all participating countries. In total, 10107 persons are included in the harmonized dataset with a mean age of 72.8 years (SD 6.1). The female/male ratio is 53.3/46.7%. Some variables were difficult to harmonize due to differences in wording and categories, differences in classifications and absence of data in some countries. The post harmonization algorithms are described in detail in harmonization guidelines attached to this paper.

**Conclusions:**

There was little evidence of agreement on the use of several core data collection instruments, in particular on the measurement of OA. The heterogeneity of OA definitions hampers comparing prevalence rates of OA, but other research questions can be investigated using high quality harmonized data. By publishing the harmonization guidelines, insight is given into (the interpretation of) all post harmonized data of the EPOSA study.

## Background

Osteoarthritis (OA) is a process or condition affecting the joint cartilage and subchondral bone and is frequently accompanied by pain, stiffness, disability and radiographic changes [1]. It is the most common cause of chronic pain in older persons and the leading cause of disability [2]. The prevalence of OA varies across countries and study populations [3,4]. A review among 29 studies from 14 countries showed a range in prevalence of knee and hip OA from 0.5 to 36% [5]. Differences in prevalence rates between countries can (partly) be attributed to differences in study population and research methods used; however, national differences such as climate or health care may also play a role. In the literature, prevalence rates have often been based on general practitioners' or other medical registries, but not all OA-patients consult a health care professional [6] and thus, these studies may underestimate the actual prevalence rates [7].

The European Project on Osteoarthritis (EPOSA) studies the personal and societal burden and its determinants of OA in the aging European population using data from five population-based cohort studies across Europe. These cohorts include not only persons with severe OA who receive treatment, but also persons with (mild) OA who have not sought care (so far). Emphasis lies on the personal consequences (such as quality of life and social participation) and societal consequences (such as health care use) of OA. Population-based studies are important as they provide data on the burden of the disease in terms of prevalence and impact on quality of life and health status, thus offering insight into the need for health care and prevention strategies.

The cohorts involved have not been set up in a standardized way and different sampling strategies have been used. The present paper provides a description of the cohorts included in the project, focuses on the post harmonization procedures to merge these cross-national data and describes the methodological challenges encountered in the process of harmonization. Our purpose is to document the degree to which these post harmonization efforts have succeeded. Furthermore, this paper describes to what extent common data collection instruments have been used and the important similarities and dissimilarities between these instruments. Post harmonization of OA, ADL limitations and social participation are described in detail to illustrate particular methodological challenges. The complete harmonization guidelines can be found in additional file 1: Harmonization guidelines.

## Methods

### Study design

The data of five existing longitudinal cohort studies are combined using post harmonization procedures. The five cohorts are population based, but vary in recruitment procedures and measurement instruments used. Table 1 provides an overview of the main characteristics of the five cohorts. The design and procedures of all five cohort studies have been approved by the Medical Ethics committee of the respective institutes.

**Table 1:** Overview of the main characteristics of the five cohorts included in EPOSA

	ActiFE	ProVA	LASA	Peñagrande	HCS
Country	Germany	Italy	Netherlands	Spain	UK

Region	Ulm	Camposampiero, Rovigo	Amsterdam, Zwolle, Oss	Peñagrande (Madrid)	Hertfordshire

Baseline year	2009	1995-1997	1992-1993	2007-2008	1999-2004

Number of follow-up cycles	0	2	5	0	2

Year of last follow-up cycle	-	2002-2004	2008	-	2007

Sample size at baseline	1506	3099	3107	814	3225

Recruitment criteria	Random selection from municipality registries, aged 65 years and older, stratified for age and sex	Random selection from health district registries, aged 65 years and older, stratified for age and sex	Random selection from municipality registries stratified for age, sex and 5 years mortality rate	Random selection from municipality registries stratified for age and sex	Participants were born during 1931-1939 in Hertfordshire, and still lived there during 1998-2003

Baseline age-range (years)	65+	65+	55-85	65+	60-72

Baseline age (years, mean (SD))	74.7 (6.5)	76.4 (7.8)	70.8 (8.8)	77 (7.6)	67.4 (1.9)

Sex (% female)	43.5	59.9	57.6	51,5	47.3

Education (% secondary education or higher)	20.4	11.8	61.1	19.8	5.2

Marital status (% married)	65.2	50.0	53.3	63.4	77.8

ActiFE: study on Activity and Function in the Elderly in Ulm, ProVA: Progetto Veneto Anziani study, LASA: Longitudinal Aging Study Amsterdam, Peñagrande: Ageing in Peñagrande, HCS: Hertfordshire Cohort Study.

### Study samples

The EPOSA project involves five cohort studies performed in five different countries. While the methodology of each cohort is described in detail elsewhere, here a brief description of their main characteristics is provided.

Germany is represented by the University of Ulm, Institute of Epidemiology and Medical Biometry and AGAPLESION Bethesda Clinic Ulm. The study on Activity and Function in the Elderly in Ulm (ActiFE-Ulm) [8] is embedded in a European funded study on the prevalence of COPD and asthma (Indicators for Monitoring COPD and Asthma - IMCA). A random sample of 7460 persons aged 65 years and over was selected from the population registers in Ulm, Neu-Ulm and Alb-Donau-Kreis. The recruitment phase started in February 2009 and finished in April 2010. In total, 1506 persons agreed to participate in the study. The primary focus is physical activity (as measured by sensor technology) and the consequences of physical activity for cognitive, emotional and social functioning.

Italy is represented by the Department of Medical and Surgical Sciences, University of Padova, and National Research Council (CNR), Aging Branch, Institute of Neuroscience, Padova. The Progetto Veneto Anziani (ProVA) study was originally designed to assess the effects of cardiovascular and osteoarticular diseases on disability [9]. Participants (65+ years, n = 3099) were living in two geographical areas of north-eastern Italy (Camposampiero and Rovigo) near the city of Padova in the Veneto region. Participants were interviewed at home, and invited to the clinic for a general physical examination and additional diagnostic tests. Finally, physicians performed a medical chart review.

The Netherlands is represented by the VU University Medical Center, EMGO Institute for Health and Care Research, Amsterdam. Data were used from the Longitudinal Aging Study Amsterdam (LASA) [10], an ongoing cohort study of predictors and consequences of changes in physical, cognitive, emotional and social functioning in older persons. In 1992/93 the first measurement cycle was completed within a random sample of older persons (55-85 years). The sample was selected from the population registers in 11 municipalities in the Netherlands and stratified for age, sex and level of urbanization. Each measurement cycle consists of a main interview, a medical interview (both face-to-face) and a self-administered questionnaire. For the current study, data were used from 1669 persons aged 65 years and older who participated in the fourth measurement cycle in 2001/2002.

Spain is represented by the Universidad Autónoma de Madrid Centro Universitario de Salud Pública. The study included is Ageing in Peñagrande [11]. Participants (65+) were selected from the Register of Health District Area in the neighbourhood of Peñagrande, which is part of the Fuencarral district in Madrid. Of the 4,244 persons of 65 years and over living in Peñagrande as of July 31, 2007, a random, age and sex stratified sample of 1250 persons was drawn of whom 1110 were eligible (88.8%) and 814 consented to participate. The baseline measurement took place in 2007-2008 and consisted of face-to-face interviews.

The United Kingdom is represented by the University of Southampton, Southampton General Hospital, MRC Lifecourse Epidemiology Unit. The Hertfordshire Cohort Study (HCS) was designed to examine the relationship between growth in infancy and the subsequent risk of common adult diseases, including osteoporosis and osteoarthritis [12]. Potential participants in HCS were born during 1931-1939 in Hertfordshire, and still lived there during the period 1998-2003 (n = 8,650). Persons who consented were visited at home where a structured questionnaire was administered (n = 3,225). Participants then attended a local morning clinic where a variety of investigations were performed (n = 2,997). In 2004/05, a follow-up clinical study was performed in East Hertfordshire (n = 966). In 2007, a postal questionnaire enquiring about clinical events over the follow-up period, was sent to all participants throughout Hertfordshire (n = 2,299). For the current study, data from the baseline measurement was used from persons who visited the clinic and who were 65 years and over (n = 1,879).

### Harmonization procedure

When various samples are used to study one particular hypothesis, the problem of heterogeneity between samples emerges. To cope with these differences, two approaches are possible: centralized analyses, which include harmonization of the datasets to create one large dataset in which the analyses are done, or coordinated independent analyses, in which the analyses are done in each dataset and the results per dataset are pooled afterwards [13]. In the current study, preference was given to centralized analyses, because this approach enables analyses of which covariates explain differences between the countries. Post harmonization guidelines were developed to overcome the differences in measurement instruments between the cohorts. The harmonization guidelines used in the Comparison of Longitudinal European Studies on Aging (CLESA) project served as an example for the current study [14].

**Figure 1:**
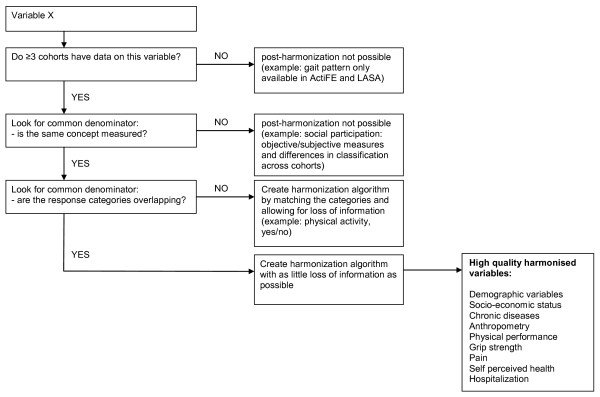
**Schematic presentation of the harmonization process for all non-OA variables**.

Figure 1 shows a schematic presentation of the harmonization procedures. Data from all cohorts were merged based on the common denominator available for each variable if at least three cohorts had the information relative to that specific measure available. If not all countries had the same detail of information, detailed information was lost in the harmonized variable. Alternatively, the same variable could be created for less than all five but more than two countries, if this led to preservation of detailed information. The cohort without detailed information was coded as having missing values on this more detailed variable. Cut-off values for categorization of variables were based on cut-off values from the literature. If no cut-off values were available, cut-off values were based on biological criteria or the distributions in the cohorts. Figure 1 also lists all harmonized variables of which the harmonization was successful (i.e. no or very little loss of information). Although information on radiographic OA and site-specific clinical OA was available in two cohorts only, it was included in the harmonized database, as these are main variables in the assessment of OA.

The involved partners sent all relevant data with variable and value labels in English to the coordinating center (VU University Medical Center, Amsterdam, the Netherlands). The coordinating center then developed the harmonized dataset in cooperation with the partners. A code book (harmonization guidelines) was made explaining how the variables were harmonized (see additional file 1: Harmonization guidelines).

### Weighting procedure

Datasets were merged, but sample sizes and age and sex distributions differed across cohorts due to differences in recruitment procedures and inclusion criteria. Sample weights were calculated to adjust for differences in recruitment procedures and inclusion criteria. The weights were calculated per sex and per five-year age category, using the following formula: W = Nexp/Nobs, with the Nobs being the number of persons in a specific age/sex category in the cohort, and Nexp being the number of persons in a specific age/sex category in the population [14]. The expected number of persons were derived from the European Standard Population [15]. Nexp for the European population were based on the numbers for 2001 (average baseline year) and included 27 countries. The weights for each of the countries were based on the numbers for that country given their specific baseline year.

## Results

### Description of main characteristics of the cohorts

Table 1 shows the main characteristics of the five cohorts that were included in the EPOSA study. The differences in recruitment criteria become evident in the differences in baseline characteristics of the five cohorts. Table 2 shows the characteristics of the specific measurement cycle of each cohort that is used in the EPOSA study. The ActiFE, ProVA, LASA and Peñagrande studies were similar regarding age distribution, however, in HCS, the mean age was approximately 10 years lower. In ActiFE and HCS the percentage of men was higher than in the other studies. Note that the years of baseline in EPOSA differ across the cohorts. For each cohort, the most recent measurement cycle that contained comprehensive information on OA was chosen as the baseline measurement in EPOSA. For example, 2001/02 was chosen as the baseline year in LASA, because this was the most recent measurement cycle in which both self-reported and general practitioners information on the presence of OA was available.

**Table 2:** Characteristics of the measurement cycles used in EPOSA

	ActiFE	ProVA	LASA	Peñagrande	HCS
Country	Germany	Italy	Netherlands	Spain	UK

Year of baseline in EPOSA	2009	1995-1997	2001-2002	2007-2008	1999-2004

Sample size					

Invited	7460	4476	2076	1110	7106

Responded	1506	3099	1691	814	2997

Response rate	19.8	69.2	81.5	73.3	42.2

Age (years, mean (SD))	74.7 (6.5)	76.3 (7.8)	76.4 (8.0)	74.8 (7.2)	65.6 (2.9)

Sex (% female)	43.5	59.9	57.6	59.1	47.3

Education (% secondary education or higher)	20.4	11.7	61.0	22.4	5.2

Marital status (% married)	65.2	49.8	46.4	64.3	77.8

ActiFE: study on Activity and Function in the Elderly in Ulm, ProVA: Progetto Veneto Anziani study, LASA: Longitudinal Aging Study Amsterdam, Peñagrande: Ageing in Peñagrande, HCS: Hertfordshire Cohort Study.

In this paper it is not possible to describe the harmonization process for all variables included in the EPOSA database. Please refer to the harmonization guidelines in additional file 1 for a detailed description of the algorithms used per variable. Here, harmonization of OA, ADL limitations and social participation is described in detail to illustrate the challenges and solutions in the harmonization process.

### Harmonization of OA

OA case definitions used in the literature vary by site of joint involvement (knee, hip, hand, generalised, unspecified) and by method (self-report, clinical diagnosis/classification, plain radiography or other imaging, combinations of these). Each of these different approaches to OA case definitions was evident in the five cohorts, highlighting the lack of accepted standard case definitions in current European studies. As OA is believed to be a collection of disorders with shared features rather than a single disease entity, several definitions of OA may be the best strategy. Thus, it was decided not to develop one harmonized variable for OA, but rather develop several OA variables (site-specific/non-specific self reported OA, site-specific/non-specific clinical OA and radiographic OA) which can be used according to specific research questions. Table 3 shows which questions or measurements were used to construct the self-reported, clinical and radiographic definitions for knee, hip, hand and non-specific OA in each of the cohorts. Note that not all definitions were available for all cohorts. However, because OA is our main variable of interest in this study, we made an exception regarding the harmonization procedure: all types of OA definitions were constructed, even when data from only two countries were present.

**Table 3:** Definitions of OA in the cohort studies included in EPOSA

Cohort					
	**ActiFE**	**ProVA^†^**	**LASA**	**Peñagrande**	**HCS^‡ ^**

**Self-reported OA**					

Knee	-	Pain or difficulty moving the knees in the last year? Positive if yes for > 1 month.	Do you have OA? If yes: Would you please tell me if you have complaints of the knee?	-	-

Hip	-	Pain or difficulty moving the hips in the last year? Positive if yes for > 1 month.	Do you have OA? If yes: Would you please tell me if you have complaints of the hip?	-	-

Hand	-	Pain or difficulty moving the hands in the last year? Positive if yes for > 1 month.	Do you have OA? If yes: Would you please tell me if you have complaints of the fingers or hand/wrist?	-	-

Non-specific	-	Positive if "yes" on any of the above	Do you have OA?	Have you had pains in the joints or bones? Did you visit a doctor for this problem? Positive if yes on both questions.	-

**Clinical OA**					

Knee	-	Judgement by physician based on physical examination and information from medical records	-	-	Told by doctor to have knee OA

Hip	-	Judgement by physician based on physical examination and information from medical records	-	-	-

Hand	-	Judgement by physician based on physical examination and information from medical records	-	-	Combination of observed Heberdens nodes by trained nurse and self-reported hand pain.

Non-specific	Has a doctor ever told you that you have or had OA/arthritis?	Positive if "yes" on any of the above	General practitioners questionnaire: Has your patient been diagnosed with OA?	Diagnosis in general practitioners' records	Positive if "yes" on any of the above

**Radiographic OA**					

Knee	-	JSN (K&L ≥ 2) + osteophytes	-	-	JSN (K&L ≥ 2) + osteophytes

Hip	-		-	-	-

Hand	-	DIP, PIP of digits 2-3, and CMC of digit 1: JSN (K&L ≥ 2) + osteophytes	-	-	DIP, PIP, CMC of digits 2-5, and IP: JSN (K&L ≥ 2) + osteophytes

ActiFE: study on Activity and Function in the Elderly in Ulm, ProVA: Progetto Veneto Anziani, LASA: Longitudinal Aging Study Amsterdam, Peñagrande: Ageing in Peñagrande, HCS: Hertfordshire Cohort Study, DIP: distal interphalangeal joint, PIP: proximal interphalangeal joint, CMC: carpo-metacarpal joint, IP: interphalangeal joint, K&L: Kellgren & Lawrence grading.

^† ^In ProVA., all x-rays were taken at the time of the clinic visit, at the same hospital using the same radiographic equipment with standardized procedures, focus to film distance 100 cm, 55 kV, 8 mA/s. Anteroposterior x-ray of the hip, and weight-bearing anteroposterior and lateral x-rays of both extended knees were performed in standing position. Standard posterior-anterior radiographs of both hands with palm flat and fingers straight were performed.

^‡ ^In HCS, weight bearing anterior-posterior and lateral semi-flexed radiographs of both knees were taken at the same hospital using the same radiographic equipment; a standard tube to film distance of 100|cm was used. Radiographs were performed at a median duration of 6|months (interquartile range (IQR) 4.8-7.2) after the clinic visit. Using the same equipment, anterior-posterior hand radiographs were obtained, with the hand placed as flat as possible.

In ProVA, persons were classified by physicians as having definite, possible or no OA in the knees, hips or hands. In the other countries, however, dichotomous definitions for clinical OA were used (yes/no). To test whether the possible-category in the ProVA data should be interpreted as either definite/yes or no, two statistical procedures were used: (1) optimal scaling technique, and (2) regression models with known OA-correlates. The optimal scaling technique used is called Princals, which is based on non-linear factor analysis [16]. This technique quantifies the distance between the scaling points (i.e. definite, possible, no). Regression analyses were done to test the associations with use of pain/anti-inflammatory drugs, physical performance, ADL limitations, and grip strength (left and right). The optimal scaling analysis and regression analyses showed smaller distances between 'definite' and 'possible' than between 'possible' and 'no' for clinical knee, hip and hand OA (data not shown). Therefore, 'possible' was interpreted as 'definite clinical OA' in the ProVA cohort.

Table 4 presents the prevalence rates for knee, hip, hand and non-specific OA per definition used. The lowest site-specific prevalence rate was found for radiographic knee OA in the UK, the highest prevalence rate was found for clinical knee OA in ProVA. In LASA and Peñagrande, prevalence rates for self-reported OA seem to be higher than for clinical OA, however, in Italy, a reverse pattern was found. The agreement between self-reported and clinical OA varied between 77 and 82%. In HCS, prevalence rates were higher for clinical than radiographic knee OA, but lower for clinical than radiographic hand OA. The agreement between clinical and radiographic OA was 64% for the hand and 79% for the knee.

**Table 4:** Prevalence rates of Osteoarthritis (OA) per site, per country and per definition

	*65-74 years old*				
	**Germany**	**Italy**	**Netherlands**	**Spain**	**UK**
	
	**Definitions based on self-report**				

Knee OA	-	19.2 (17.1-21.3) n = 1412	23.6 (20.5-26.7) n = 706	-	-

Hip OA	-	14.1 (12.3-15.9) n = 1412	17.4 (14.6-20.2) n = 706	-	-

Hand OA	-	15.7 (13.8-17.6) n = 1411	19.1 (16.2-22.0) n = 706	-	-

Non-specific	-	32.3 (29.9-34.7) n = 1411	41.4 (37.8-45.0) n = 735	56.2 (50.8-61.6) n = 326	-

	**Definitions based on clinical judgement**				

Knee OA	-	36.7 (34.2-39.2) n = 1434	-	-	18.6 (16.0-21.2) n = 878

Hip OA	-	22.9 (20.7-25.1) n = 1434	-	-	-

Hand OA	-	33.1 (30.7-35.5) n = 1433	-	-	23.1 (21.1-25.1) n = 1678

Non-specific	44.7 (41.4-48.1) N = 830	54.6 (52.0-57.2) n = 1433	23.7 (20.0-27.4) n = 510	43.6 (38.2-49.0) n = 328	29.3 (27.1-31.4) n = 1688

	**Definitions based on radiographic information**				

Knee OA	-	8.6 (6.4-10.8) n = 647	-	-	15.1 (11.4-18.8) n = 360

Hip OA	-	3.5 (2.1-4.9) n = 641	-	-	-

Hand OA	-	31.0 (26.3-35.7) n = 375	-	-	28.1 (19.3-36.9) n = 100

	***75+ years old***				

	**Germany**	**Italy**	**Netherlands**	**Spain**	**UK**
	
	**Definitions based on self-report**				

Knee OA	-	24.3 (22.1-26.5) n = 1436	31.4 (28.0-34.8) n = 696	-	-

Hip OA	-	18.9 (16.9-20.9) n = 1436	19.1 (16.2-22.0) n = 696	-	-

Hand OA	-	21.9 (19.8-24.0) n = 1436	25.9 (22.6-29.2) n = 697	-	-

Non-specific	-	40.4 (37.9-42.9) n = 1433	53.5 (49.9-57.1) n = 758	65.5 (61.2-69.8) n = 460	-

	**Definitions based on clinical judgement**				

Knee OA	-	50.8 (48.4-53.2) n = 1672	-	-	-

Hip OA	-	36.1 (33.8-38.4) n = 1672	-	-	-

Hand OA	-	44.1 (41.7-46.5) n = 1674	-	-	-

Non-specific	47.9 (44.1-51.7) n = 670	69.6 (67.4-71.8) n = 1672	40.5 (36.4-44.6) n = 549	56.6 (52.1-61.1) n = 468	-

	**Definitions based on radiographic information**				

Knee OA	-	17.7 (14.7-20.7) n = 610	-	-	-

Hip OA	-	7.4 (5.3-9.5) n = 584	-	-	-

Hand OA	-	55.0 (49.8-60.2) n = 358	-	-	-

Presented are the prevalence rates and 95% confidence intervals (95%CI) for site-specific and non-specific OA per country and per definition used. 95%CI was calculated as: p ± 1.96 * SE in which SE = √ (p (1 - p)/n) and p = proportion. Results were weighted for age and sex in the European Standard Population as of January 1, 2001, and stratified for age.

### Harmonization of ADL limitations

In all five cohorts, a variety of Activity of Daily Living (ADL) variables were assessed in structured interviews (Table 5). First it was investigated which items were available in all cohorts. The item "do you have difficulty with walking up and down from a staircase?" was available in all cohorts and therefore included in the harmonized ADL score. In the LASA study, the item "do you have difficulty with taking a shower or bath?" was not available, but a proxy was used from the quality of life index score of the EQ-5D: "how much difficulty do you have with washing or dressing yourself?" Subsequently, this item was available in all cohorts and included in the harmonized ADL score. Second, it was investigated which items were available in most cohorts, which resulted in the selection of seven items in total (see Table 5). Of these seven items, five items were used and per item 0 to 2 points were assigned according to the level of difficulty with that activity (0: no difficulty, 1: with difficulty, 2: unable to do alone). Thus, an ADL score ranging from 0-10 was made in all cohorts, but the actual items used within this score partly differ across cohorts. However, we tried to match the items as closely as possible according to the underlying physical demands. For example, the ActiFE and LASA studies did not have the item "Do you have difficulty with using your fingers to catch or handle small objects?". Instead, the item "Do you have difficulty cutting your toenails?" was used, because this activity also requires fine motor skills of the hands.

**Table 5:** Available items of Activity of Daily Living in each cohort

ADL item	ActiFE	ProVa	LASA	Peñagrande	HCS
**Walk up/down stairs**	+	+	+	+	+
**Cut your toe nails**	+	+	+	-	+
**Dress/undress**	+	+	+	+	-
**Sit down/rise from a chair**	+	+	+	+	-
Walk outside without rest	+	-	+	-	-
Use own/public transportation	+	-	+	+	-
**Take shower/bath**	+	+	+	+	+
Light housework	+	-	-	-	-
Walk into room	-	+	-	+	-
Eating/using cutlery	-	+	-	+	-
Use toilet	-	+	-	+	-
**Raising arms above head**	-	+	-	+	+
**Use fingers to catch/handle small things**	-	+	-	+	-
Running to catch a bus	-	-	-	+	-
Heavy housework	-	-	-	+	-
Preparing a hot meal	-	-	-	+	+
Shopping/carrying a bag/weight > 5 kg	+	+	-	+	+

ActiFE: study on Activity and Function in the Elderly in Ulm, ProVA: Progetto Veneto Anziani study, LASA: Longitudinal Aging Study Amsterdam, Peñagrande: Ageing in Peñagrande, HCS: Hertfordshire Cohort Study. The items in bold were used to construct the variable "number of ADL limitations" in EPOSA.

### Harmonization of social participation

All five cohorts had some information on social participation. In the ActiFE study the Lubben Social Network Scale was used, which assesses social isolation in older adults by measuring perceived social support received by family and friends. Two examples of items of this questionnaire are "How many relatives do you see or hear from at least once a month?" and "How many relatives do you feel at ease with that you can talk about private matters?". If social participation is regarded as an objective measurement of social activity, it is not possible to include data from ActiFE, as the questionnaire measures self perceived social support rather than engagement in social activities. In ProVA, Peñagrande, LASA and HCS, the frequency of participation in social activities was measured. In HCS the social activity score was derived from the social health questionnaire which includes the participation and frequency of 13 activities, such as positions of office or visiting friends. In Peñagrande, only two questions were asked regarding the frequency of caring for sick persons and caring for children. In LASA, respondents were asked whether they were involved in social organizations such as a political party or sport club and how often they participated in activities or meetings of these organizations. Although the frequency of social activities was assessed in four cohorts, the response categories varied: e.g. in ProVA the number of weekly hours devoted to social activities was asked, while in HCS the response categories were "weekly", "monthly" or "less often". Thus, the number and types of activities and the response categories differ greatly across the cohorts. It was therefore concluded that the social participation variables from the different cohorts were too heterogeneous to be harmonized.

## Discussion

As the percentage of older persons is increasing rapidly across the Western world, the prevalence of OA is expected to rise [2]. So far, OA has received little attention from clinicians and health care organisations. OA has mainly been studied clinically in a selected patient population. However, prevalence rates, course and consequences of OA in the general population are still largely unknown [7]. World-wide large variations exist in treatment guidelines and timing of joint replacement surgeries [17,18]. The EPOSA study was initiated to add to the insight into the correlates of OA and the role of differences in geographics, socio-economic status, and health care policies between European countries. Since OA cannot be cured, insight into the physical, mental and social consequences of OA is important. Such consequences may be more important outcomes of treatment than disease severity. However, there is a gap in the literature on these consequences in the general population. The EPOSA study aims to bridge this gap. This information will help to improve guidelines for the treatment of OA and subsequently the quality of life in OA patients.

In this paper, the harmonization procedures are described that are used in the European Project on Osteoarthritis (EPOSA). This study combines data of existing cohort studies across five European countries varying in climate, socio-economic status, life style, and health care policies. Across the cohorts, different measurement instruments were used and post harmonization algorithms were needed to merge the datasets and enable statistical analyses that allow testing cross-country differences.

Due to the lack in standardization of the definition of OA, it was impossible to harmonize OA into one variable of OA. However, OA is believed to be a collection of disorders with shared features rather than a single disease entity, suggesting that it is appropriate to use several definitions of OA. Different definitions were constructed, based on three sources of information, i.e. self-report, clinical diagnosis and radiography. Higher rates of knee, hand and hip OA were found, for self-reported definitions than for the clinical definitions and radiography. These findings suggest that the prevalence rates of OA are higher for less specific definitions (e.g. pain or self-report) than for more specific definitions (clinical judgement by a rheumatologist). The only exception is the ProVA study, in which the self-reported prevalence rates were lower than the clinical prevalence rates. This may be explained by the fact that we interpreted "possible clinical OA" as having OA, which may have led to an overestimation. If the "possible" category is interpreted as not having OA, the prevalence rates for clinical OA are lower than for self-reported OA (data not shown). In contrast to our findings, a recent review study showed that prevalence rates were higher when radiographic OA definition was used compared to symptomatic or self-reported OA definitions (Pereira, 2011). This study included population based studies as well as hospital based studies, which makes it difficult to compare these results with the results of our study. The study also showed the difficulty in drawing conclusions on pooled prevalence rates due to large differences in study design, definition of OA and measurement of OA.

A limitation of this study might be the lack of radiographic OA data in most cohorts (only available in ProVA and HCS), as radiographic OA is still seen as the standard case definition of OA in many epidemiological studies [19]. Also the widely used American College of Rheumatology (ACR) criteria include both clinical assessment and radiography in the definition of knee, hip and hand OA [20-22]. These criteria are developed for patients who report to their doctor with pain. The EPOSA study however, is completely population based, including both OA patients (who do not always seek care) and healthy persons. Performing radiography in population based studies is not common and often not feasible.

Despite the harmonization efforts, important differences remain in the interpretation of the OA-definitions for each EPOSA cohort. The number of joints included in the non-specific OA definitions varied (two joints in HCS, three joints in ProVA, and all joints in LASA and Peñagrande) and the source of information varied (e.g. clinical definitions were based on physical examinations carried out as part of the study in ProVA, on information available in medical records in LASA and Peñagrande, and on self-report of being diagnosed with OA by a physician in HCS and ActiFE, and self-reported OA was based on the question "do you have OA?" in LASA and on reported pain or difficulty moving the joints (Peñagrande and ProVA). These differences in definitions hamper cross-country comparisons of prevalence rates. The interpretation of the definitions between countries is too diverse and pre harmonization is needed to reliably study cross-national differences in prevalence rates of OA. In the literature, much effort has been devoted to developing a standard definition of OA for epidemiologic studies that includes symptoms, disability, and joint structural disease [23]. A difficulty in establishing a single definition is that although there is some correlation between radiographic disease severity and both symptoms and disability, the relationships are not as strong as expected [24,25]. The results of these studies suggest that OA is a collection of disorders with shared features rather than a single disease entity, resulting in different OA phenotypes. In our study we tried to harmonize different OA variables into three definitions (self-reported, clinical and radiographic OA), enabling studying different phenotypes of OA, depending on specific research questions.

Recently, recommendations for standardization of radiographic OA and symptomatic OA were given by researchers of the Translational Research in Europe Applied Technologies for Osteo-Arthritis (TREAT-OA) consortium, a large study on genetic and biochemical risk factors of OA [26]. Consensus was reached that radiographic knee OA should be defined with the original Kellgren & Lawrence score "definite osteophytes and possible joint space narrowing", which is in agreement with our study. Radiographic hip OA was defined as "at least definite joint space narrowing", and no consensus was reached for the definition of radiographic hand OA. It was also not possible to standardize symptomatic OA since all studies defined symptomatic OA differently. The authors suggest including pain, clinical assessment of OA as well as radiographic data in this definition [26]. Efforts in the development of knee OA definitions for use in epidemiological studies have led to the EULAR-recommendations [27], however, the information needed for these definitions (symptoms: pain, morning stiffness and functional limitations and signs: crepitus, restricted movement and bony enlargement) is not yet commonly measured in existing cohort studies. Until common definitions are available in cohort studies, post harmonization procedures, although not ideal, are the only available option for cross-cohort comparison of prevalence rates. Unfortunately, the cross-national EPOSA dataset is not well suited for research on the prevalence of OA. However, the dataset can be used to study associations between OA and risk factors or consequences in one or more cohorts that use a similar OA definition.

Post harmonization of other instruments other than OA measures was also problematic. Although a common ADL limitations measure was constructed, it has to be tested whether this measure is a valid and reliable instrument. Loss of information was especially great regarding social participation. Unfortunately, these data could not be harmonized because of heterogeneity of the social activity questionnaires across cohorts.

The measurement instruments used in each of the cohorts and for all variables were carefully selected by each of the cohorts and validated instruments were used if available. However, it is unclear to what degree the harmonized variables are valid. Validating all constructed variables is not feasible. To compensate for this, we are publishing the harmonization guidelines to be transparent about the data harmonization. In future papers the effect of harmonization on the validity of the main variables used will be discussed in the concerning papers.

Fortunately, many other variables were successfully harmonized and include presence of other chronic diseases, anthropometric measures, physical performance, grip strength, pain, self perceived health and hospitalization. Although the intention of the harmonization was to study the prevalence rates of OA across countries and to focus on personal and societal consequences of OA, we cannot pursue these research questions with the current harmonized OA data. On the other hand, it is very well possible to study risk factors and consequences of OA in a part of the cohorts (based on the research questions and OA definitions available in the cohorts) and to study other non-OA-related research questions using high quality harmonized EPOSA data.

Post harmonization of epidemiologic studies has become more common in the past 20 years [28]. It is of great importance to address issues that arise when original data are being harmonized. When attempting post harmonization of data from existing cohort studies the challenges described in this paper are likely to occur. Although the cohorts that participate in the EPOSA study use common data collection instruments, still large differences in many variables existed due to differences in wording and categories, differences in classifications or absence of data. When harmonization leads to too much loss of information, for example in social participation, analysis can be done per cohort. Overall estimates can be obtained by pooling the results. However, this approach hampers cross-national comparisons and difficulty with the interpretation of the results remains. The results of this current paper show the urgency for more agreement on common data collection instruments in the design stage of cohort studies rather then retrospectively to facilitate pooling of data and cross-national comparison.

Despite these issues, the EPOSA dataset provides a unique opportunity to study various research questions in general populations of older persons across Europe. The cross-national nature of the study provides for a large number of older persons in the analyses and large variation across cohorts, resulting in sufficient power to draw conclusions with respect to associations between variables. The harmonized dataset allows for analysis on the individual level, and stratified analyses allow for studying cross-nation differences. In addition, direct replication of findings across countries is possible. Four of the cohorts participating in the EPOSA study provide follow-up data, enabling longitudinal data analyses.

Despite the extensive harmonization procedure, some variables, including OA, may continue to be difficult to compare across countries, and interpretation of findings may require particular attention. These aspects will be considered carefully by all involved investigators, and potential biases in the cross-national comparisons will be discussed in each future article.

## Conclusions

This paper provides the harmonization procedures on OA and other selected variables of the EPOSA project. This paper shows the degree of success of the post harmonization attempts that have been made. Given the heterogeneity within the OA definitions, this dataset is less suitable to study prevalence rates of OA. However, the dataset is suitable for studying other research questions using high quality harmonized data. Furthermore, it is recommended that researchers reach agreement on data collection instruments in the design stage of cohort studies to facilitate successful pooling of data.

## Competing interests

The authors declare that they have no competing interests.

## Authors' contributions

GP and LS drafted the manuscript. All authors contributed to the conception and design of the study. All authors read and corrected draft versions of the manuscript and approved of the printed version.

## Pre-publication history

The pre-publication history for this paper can be accessed here:

http://www.biomedcentral.com/1471-2474/12/272/prepub

## Supplementary Material

Additional file 1 **Harmonization guidelines**. This file provides all harmonized variables of the EPOSA study in detail. Click here for file
