# Supplementary material for: European Project on Osteoarthritis (EPOSA): methodological challenges in harmonization of existing data from five European population-based cohorts on aging
Source: BMC Musculoskelet Disord. 2011 Nov 28;12:272. doi: 10.1186/1471-2474-12-272 (PMC3257208; doi:10.1186/1471-2474-12-272)
Supplement: Additional file 1 — Harmonization guidelines. This file provides all harmonized variables of the EPOSA study in detail. [file 1471-2474-12-272-S1.PDF]

ADDITIONAL FILE 1: HARMONIZATION GUIDELINES

GENERAL VARIABLES

STUDY	QUESTION (variable name)	VALUE	EPOSA CODE
	**Country**	(country)	1. Germany<br>2. Italy<br>3. Netherlands<br>4. Spain<br>5. UK
	**ID**	(ID)	Germany →10000000+ID<br>Italy → 20000000+ID<br>Netherland → 30000000+respnr<br>Spain → 40000000+hc1<br>UK → 50000000+abserno
	**Proxy**	(proxy)	0. proxy<br>1. respondent
Germany	Who has answered the questionnaire?<br>At the end of each of the three visits<br>(IV1N410, IV2N657a, IV3N895)	1. only participant<br>2. mostly participant<br>3. mostly relatives/friends<br>4. other situation	1,2 → 1<br>3,4 → 0
Italy	Type of interview (int)	0. direct<br>1. indirect	0 → 1<br>1 → 0
Netherlands	Not available	all face-to-face data come from respondent	1
Spain	Type of interview (proxy)<br>*this variable was not provided*	1. no<br>2. yes	1 → 1<br>2 → 0
UK	Not available		1

	**Weights – European standard population, 2001**	(wEU65t74)<br>(wEU75p)	Range: 0- highest
Germany			weights by sex and age (65+) according to the European Standard Population, 2001 (http://epp.eurostat.ec.europa.eu/portal/page/portal/population/data/database). Per 5-year age/sex category: wEU01=N/n in which N=expected sample size (EU standard population), and n=observed sample size (sample size cohorts). We chose 2001 as reference year, because this was the median year of all baseline interviews across the cohorts.<br>Since there are no participants in HCS above the age of 72, it was not possible to compute weights for 65+. Therefore, weights were computed for two age-categories:<br>- 65-74 years including all cohorts<br>- 75+ years for It, NL, Sp<br>As a result, weighted analysis can only be done stratified for age.
Italy			
Netherlands			
Spain			
UK			
	**Weights – per country**	(w….)	Range: 0- highest
Germany		(wactife09)	wactife09=N/n<br>N=expected sample size acc to German standard population, 2009; n=observed sample size in Actife, age-range 65+
Italy		(wprova97)	wprova97=N/n<br>N=expected sample size acc to Italian standard population, 1997; n=observed sample size in Prova, age-range 65+
Netherlands		(wlasa01)	wlasa01=N/n<br>N=expected sample size acc to Dutch standard population, 2001), and n=observed sample size, age-range 65+

Spain		(wpeña08)	wpeña08=N/n<br>N=expected sample size acc to Spanish standard population, 2008; n=observed sample size in Peñagrande, age-range 65+
UK		(whcs02)	Whcs02=N/n<br>N=expected sample size UK standard population, 2002; n=observed sample size HCS, age-range 65+

OSTEOARTHRITIS

STUDY	QUESTION (variable name)	VALUE	EPOSA CODE
	**Self-reported knee OA**	(srknee)	-1. missing<br>0. no<br>1. yes
Germany	Not available		missing
Italy	Symptomatic arthrosis/arthritis of the knee reported by the patient (ARG01)	1. yes<br>2. no	1 → 1<br>2 → 0
Netherlands	Do you have osteoarthritis (erheum01)	-5. not available<br>0. no, never<br>1. no, previous yes<br>2. yes, previous no<br>3. yes, previous yes	-5 → -1<br>0 → 0<br>1 → 0<br>2 → 1<br>3 → 1
Spain	Not available		missing
UK	Not available		missing
	**Self-reported hip OA**	(srhip)	-1. missing<br>0. no<br>1. yes
Germany	Not available		missing
Italy	Symptomatic arthrosis/arthritis of the hip reported by the patient (ARA01)	1. yes<br>2. no	1 → 1<br>2 → 0
Netherlands	Do you have osteoarthritis (erheum01)<br><br>Would you please tell me if you have complaints of the hip (erheum8h)	-5. not available<br>0. no, never<br>1. no, previous yes<br>2. yes, previous no<br>3. yes, previous yes<br><br>-2 not available<br>1. not mentioned<br>2. mentioned	If erheum01≥2 and erheum8h=2 → 1<br>If erheum01<2 → 0<br>If erheum01≥2 and erheum8h=-2 → -1<br>If erheum01=-5 → -1
Spain	Not available		missing

UK	Not available		missing
	**Self-reported hand OA**	(srhand)	-1. missing<br>0. no<br>1. yes
Germany	Not available		missing
Italy	Symptomatic arthrosis of the hand reported by the patient (ARM01)	1. yes<br>2. no	1 → 1<br>2 → 0
Netherlands	Do you have osteoarthritis (erheum01)<br><br><br><br><br><br>Would you please tell me if you have complaints of …..<br>- the fingers (erheum8a)<br>- hand/wrist (erheum8b)	-5. not available<br>0. no, never<br>1. no, previous yes<br>2. yes, previous no<br>3. yes, previous yes<br><br>-2 not available<br>1. not mentioned<br>2. mentioned	If erheum01≥2 and (erheum8a=2 or erheum8b=2) → 1<br>If erheum01<2 → 0<br>If erheum01≥2 and (erheum8a=-2 and erheum8b=-2) → -1<br>If erheum01<-5 → -1
Spain	Not available		missing
UK	Not available		missing
	**Self-reported non-specific OA**	(srnonsp)	-1. missing<br>0. no<br>1. yes
Germany	Not available		missing
Italy			If yes on srknee, srhand or srhip → 1<br>If no on srknee, srhand and srhip → 0
Netherlands	Do you have osteoarthritis (erheum01)	-5. not available<br>0. no, never<br>1. no, previous yes<br>2. yes, previous no<br>3. yes, previous yes	If (erheum01>=2) → 1<br>If (erheum01<2) → 0<br>If (erheum01<0 or sysmis(erheum01)) → -1
Spain	Have you had pains in the joints or bones? (OA)	1. yes<br>2. no<br>9. don't know	If (OA=1 and OA_diagnosis~=2) → 1<br>If (OA=2 or (OA=1 and OA_diagnosis=2)) → 0<br>If (OA=9) → -1

	Diagnostic in medical record (OA_diagnosis)	1. OA<br>2. other musculoskeletal diagnosis<br>9. no musculoskeletal diagnosis	
UK	Not available		missing
**STUDY**	**QUESTION (variable name)**	**VALUE**	**EPOSA CODE**
	**Clinical knee OA**	(clknee)	-1. missing<br>0. no<br>1. yes
Germany	Not available		missing
Italy	Clinical judgement of the doctor: diagnosis of symptomatic arthrosis of the knees (ARG12)	1. definite<br>2. possible<br>3. no	1 → 1<br>2 → 1<br>3 → 0
Netherlands	Not available		missing
Spain	Not available		missing
UK	Told to have knee OA by doctor (kqoadoc)	0. no<br>1. yes<br>2. don't know	0 → 0<br>1 → 1<br>2 → -1
**STUDY**	**QUESTION (variable name)**	**VALUE**	**EPOSA CODE**
	**Clinical hip OA**	(clhip)	-1. missing<br>0. no<br>1. yes
Germany	Not available		missing
Italy	Clinical judgement of the doctor: diagnosis of arthrosis of the hip (ARA11)	1. definite<br>2. possible<br>3. no	1 → 1<br>2 → 1<br>3 → 0
Netherlands	Not available		missing
Spain	Not available		missing
UK	Not available		missing
**STUDY**	**QUESTION (variable name)**	**VALUE**	**EPOSA CODE**

	**Clinical hand OA**	(clhand)	-1. missing<br>0. no<br>1. yes
Germany	Not available		missing
Italy	Clinical judgement of the doctor: diagnosis of symptomatic arthrosis of the hands (ARM10)	1. definite<br>2. possible<br>3. no	1 → 1<br>2 → 1<br>3 → 0
Netherlands	Not available		missing
Spain	Not available		missing
UK	Hand observation (left/right):<br>- heberdens nodes (heheber)	0. no<br>1. yes	0 → 0<br>1 → 1
**STUDY**	**QUESTION (variable name)**	**VALUE**	**EPOSA CODE**
	**Clinical non-specific OA**	(clnonsp)	-1. missing<br>0. no<br>1. yes
Germany	Has a doctor ever told you that you have or had arthrosis/arthritis? (IV1N114)	1. no<br>2. yes, still causes impairment<br>3. yes, no impairment anymore	1 → 0<br>2 → 1<br>3 → 1
Italy	A doctor said he has arthrosis (AR12)	1. yes<br>2. no<br>9. don't know	Two definitions are possible:<br>*1. Based on AR12*<br>1 → 1<br>2 → 0<br>9 → -1<br>*2. Based on summing clknee, clhip and clhand:*<br>If (clknee=1 or clhip=1 or clhand=1) → 1<br>If (clknee=0 and clhip=0 and clhand=0) → 0
Netherlands	General Practitioner questionnaire: Has your patient been diagnosed with OA? (egoart)	0. no<br>1. yes	0 → 0<br>1 → 1
Spain	Diagnosis in medical record ?<br>(OA_diagnosis)	1. OA<br>2. other musculoskeletal diagnosis<br>9. no musculoskeletal diagnosis	1 → 1<br>2,9 → 0
UK			If (clknee=1 or clhip=1) → 1

			If (clknee=0 and clhip=0) → 0
**STUDY**	**QUESTION (variable name)**	**VALUE**	**EPOSA CODE**
	**Radiographic knee OA**	(xrknee)	-1. missing<br>0. no<br>1. yes
Germany	Not available		missing
Italy	joint space narrowing (k&l grading) medial and lateral:<br>- right (kjsnml, kjsnll)<br>- left (kjsnmr, kjsnlr)<br><br>Femoral/tibial medial/lateral osteophytes<br>- right (kostfml, kosttml, kostfll, kosttll)<br>- left (kostfmr, kosttmr, kostflr, kosttlr)	Range: 0-3<br><br><br><br><br>Range: 0-3	If (kjsnml>=0 and kjsnmr>=0) xrknee=0.<br>If (missing on any of the variables) xrknee=-1.<br>If (kjsnml>=2 or kjsnmr>=2 or kjsnll>=2 or kjsnlr>=2) and (kostfml>=1 or kostfmr>=1 or kosttml>=1 or kosttmr>=1 or kostfll>=1 or kostflr>=1 or kosttll>=1 or kosttlr>=1) xrknee=1.
Netherlands	Not available		missing
Spain	Not available		missing
UK	knee x-ray: anterior-posterior tibiofemoral joint k&l grading<br>- right (knxrtkl)<br>- left (knxltkl)<br><br>knee x-ray: ap right tfj medial osteophytes<br>- right (knxrmost)<br>- left (knxlmost)<br><br>knee x-ray: ap right tfj lateral osteophytes<br>- right (knxrlost)<br>- left (knxllost)	Range: 0-4<br><br><br><br><br>0. no<br>1. yes	If (knxrtkl>=0 and knxltkl>=0) → 0<br>If (knxrtkl>=2 and (knxrlost>=1 or knxrmost>=1)) or (knxltkl>=2 and (knxllost>=1 or knxlmost>=1)) → 1
**STUDY**	**QUESTION (variable name)**	**VALUE**	**EPOSA CODE**
	**Radiographic hip OA**	(xrhip)	-1. missing<br>0. no<br>1. yes

Germany	Not available		missing
Italy	Hip x-ray: joint space narrowing (K&L grading) superior/axial<br>- left (hjsnsl, hjsnal)<br>- right (hjsnsr, hjsnar)<br><br>Osteophytes: acetabular/femoral<br>- left (hostal, hostfl)<br>- right (hostar, hostfr)	Range: 0-3<br><br><br><br><br>Range: 0-3	If (hjsnsl>=0 and hjsnsr>=0) xrhip=0.<br>If (missing on any of the variables) xrhip=-1.<br>If (hjsnsl>=2 or hjsnsr>=2 or hjsnal>=2 or hjsnar>=2) and (hostal>=1 or hostar>=1 or hostfl>=1 or hostfr>=1) xrhip=1.
Netherlands	Not available		missing
Spain	Not available		missing
UK	Not available		missing
**STUDY**	**QUESTION (variable name)**	**VALUE**	**EPOSA CODE**
	**Radiographic hand OA**	(xrhand)	-1. missing<br>0. no<br>1. yes
Germany	Not available		missing
Italy	Hip x-ray: joint space narrowing (K&L grading) dip/pip of T2 and T3 and cmc<br>- left (dipjsnl3, dipjsnl2, pipjsnl3 pipjsnl2, cmcjsnl)<br>- right (dipjsnr3, dipjsnr2, pipjsnr3 pipjsnr2, cmcjsnr)<br><br>Osteophytes: dip/pip of T2 and T3 and cmc<br>- left (dipostl3, dipostl2, pipostl3, pipostl2, cmcostl)<br>- right (dipostr3, dipostr2, pipostr3, pipostr2, cmcostr)<br><br>Rheumatoid arthritis (yes/no) (mra)	Range: 0-3<br><br><br><br><br><br><br>Range: 0-3<br><br><br><br><br><br>0. no<br>1. yes	If (dipjsnl3>=0 and dipjsnr3>=0) or (mra=1) xrhand=0.<br>If (missing on any of the variables) xrhand=-1.<br>If (dipjsnl3>=2 or dipjsnl2>=2 or dipjsnr3>=2 or dipjsnr2>=2 or pipjsnl3>=2 or pipjsnl2>=2 or pipjsnr3>=2 or pipjsnr2>=2) and (dipostl3>=1 or dipostl2>=1 or dipostr3>=1 or dipostr2>=1 or pipostl3>=1 or pipostl2>=1 or pipostr3>=1 or pipostr2>=1) xrhand=1.
Netherlands	Not available		missing

Spain	Not available		missing
UK	handxray: joint space narrowing<br>- dip (hxrldipj, hxrrdipj, hxrmdipj, hxridipj, hxlldipj, hxlrdipj, hxlmdipj, hxlidipj)<br>- pip (hxrlpipj, hxrrpipj, hxrmpipj, hxripipj, hxllpipj, hxlrpipj, hxlmpipj, hxlipipj)<br>- ip (hxrtipj, hxltipj)<br>- cmc (hxrtcmcj, hxltcmcj)<br><br>handxray: osteophytes<br>- dip (hxrldipo, hxrrdipo, hxrmdipo, hxridipo, hxlldipo, hxlrdipo, hxlmdipo, hxlidipo)<br>- pip (hxrlpipo, hxrrpipo, hxrmpipo, hxripipo, hxllpipo, hxlrpipo, hxlmpipo, hxlipipo)<br>- ip (hxrtipo, hxltipo)<br>- cmc (hxrtcmco, hxltcmco)	Range: 0-4<br><br><br><br><br><br><br><br>Range: 0-3	If jsn>=2 and osteophytes>=1 in any of the joints 1 → 1<br>If jsn<2 and osteophytes=0 in all of the joints → 0

DEMOGRAPHIC VARIABLES

STUDY	QUESTION (variable name)	VALUE	EPOSA CODE
	**Date of birth**		
Germany	Date of birth: day, month, year (IV1N008_01, IV1N008_02, IV1N008_03)		
Italy			
Netherlands	Birthday (geb_dat)	SPSS date format	
Spain	Not available		
UK	Date of birth: day (hdobdd)<br>Date of birth: month (hdobmm)<br>Date of birth: year (hdobyy)		
	**Age at baseline**	(age)	Range: 50-highest (continuous)
Germany	Years between date of interview and date of birth	range: 65 - highest	
Italy	Age (age)	range: 65-highest	
Netherlands	The age at baseline depends on the year taken as baseline. (eage)	range: 55.0-highest	Copy and rounded of to the nearest integer
Spain	Age (age)	range: 65-highest	
UK	Years between date of birth (hdobdd, hdomm, hdoyy) and date of interview : -HCS ('99-'04): hdoidd, hdoimm, hdoiyy<br>-MFSU ('04-'05): fqdoidd, fqdoimm, fqdoiyy	range: 60-highest	
	**Year of baseline interview**	(baseline)	range: 1995-2009
Germany			Copy year
Italy	Date of interview (DTinter)	dd.mm.yy	Copy year
Netherlands	Date of interview (t5_dat)	Spss date format: in days from 1-1-1582<br>→ recoded into actual dates (edateint)	Copy year
Spain			Copy year
UK	HHQ: year of interview	yyyy	Copy year

	**Sex**	(sex)	0. Male<br>1. Female
Germany	Sex (IV1N007)		
Italy	Sex (sex)	1. Male<br>2. Female	1→0<br>2→1
Netherlands	Sex (sex)	1. Male<br>2. Female	1→0<br>2→1
Spain	Sex (sex)	0. Male<br>1. Female	0→0<br>1→1
UK	Sex (absex)	1. Male<br>2. Female	1→0<br>2→1
	**Urbanisation grade**	(urban)	-1. missing<br>1. rural (<300 p/km^2, <5000 inhabitants)<br>2. intermediate (5000-30000 inhabitants)<br>3. urban (>300 p/km^2, >5000 inhabitants)
Germany	Not available as variable. Can be differentiated by rural and urban area only.		
Italy	The urbanisation grade for all participants is urban.		3
Netherlands	Urbanisation grade per municipality (CBS 1971) (burb_71c)	1. A3 (rural, <300 p/km^2, <5,000 inh)<br>2. A4 (rural, <300 p/km^2, <5,000 inh)<br>3. B1 (intermediate, <5,000 inh)<br>4. B2 (intermediate, <20,000 inh)<br>5. B3 (intermediate, <30,000 inh)<br>6. C1 (urban, >300 p/km^2, <10,000 inh)<br>7. C2 (urban, >300 p/km^2, <30,000 inh)<br>8. C3 (urban, >300 p/km^2, <50,000 inh)<br>9. C4 (urban, >300 p/km^2, <100,000)<br>10. C5 (urban, >100,000)	1,2 → 1<br>3,4,5 → 2<br>6,7,8,9,10 →3
Spain	All participants live in one region of Madrid - Peñagrande		All → 3
UK	Not available		All → -1
	**Residence**	(residence)	-1. missing<br>0. community-dwelling<br>1. institutionalized

Germany	What kind of house do you live in? (IV3N877)	1. one family house<br>2. two family house/row house<br>3. apartment in apartment building	1→0<br>2→0<br>3→0
Italy	Place of interview (residencepr)	1. home of the subject<br>2. home of a relative<br>3. hospital<br>4. nursing home or rest<br>5. other	1→0<br>2→0<br>3→0<br>4→1<br>5→0
Netherlands	Housing type (ehindep)	-3. no observation<br>-2. no answer<br>-1. unknown<br>1. independent<br>2. residential home<br>3. nursing home - somatic<br>4. nursing home – psycho geriatric<br>5. hospital<br>6. psychiatric hospital<br>8. monastry	-3,-2,-1→-1<br>1→0<br>2→0<br>3→1<br>4→1<br>5→1<br>6→1<br>8→0
Spain	To live in residence (residence)	0. no residence<br>1. yes residence	0→0<br>1→1
UK	Residence (fqresid)	1. your own home(owner)<br>2. rented accommodation<br>3. a residential home<br>4. a nursing home<br>5. other(please specify)	1→0<br>2→0<br>3→1<br>4→1<br>5→0 (except for granny flat→1)
	**Race/ethnicity**	(ethnicity)	-1. missing<br>0. Caucasian<br>1. non-caucasian
Germany	Not available	All caucasian	All 0
Italy	Race (raceprova)	All Caucasian	All 0
Netherlands	Ethnic identification (aethnic)	-2. born Neth+nat=Neth<br>1. Dutch/Netherlands<br>2. Arubian<br>3. Antillian<br>4. Chinese	-2→0<br>1→0<br>2→1<br>3→1<br>4→1

		5. Greece<br>6. Italian<br>7. Yugoslavian<br>8. Capeverdian<br>9. Moroccan<br>10. Moluccan<br>11. Portuguese<br>12. Spanish<br>13. Surinam<br>14. Tunisian<br>15. Turkish<br>16. other<br>17. Indonesian<br>18. Iranian<br>19. American<br>20. European<br>21. Polish<br>22. French<br>23. Indian<br>24. German<br>25. English	5→0<br>6→0<br>7→0<br>8→1<br>9→1<br>10→1<br>11→0<br>12→0<br>13→1<br>14→1<br>15→1<br>16→1<br>17→1<br>18→1<br>19→0<br>20→0<br>21→0<br>22→0<br>23→1<br>24→0<br>25→0
Spain	Not available		All -1
UK	Not available		All -1
	**Marital status**	(marital)	-1. missing<br>0. not married<br>1. married<br>2. widowed<br>3. divorced/separated
Germany	Marital status (IV1N020)	1. married<br>2. single/unmarried<br>3. divorced<br>4. widowed<br>5. living apart	1 → 1<br>2 → 0<br>3 → 3<br>4 → 2<br>5 → 3
Italy	Marital Status (MaritalStatus)	1. Never married<br>2. married/cohabiting	1 → 0<br>2 → 1

		3. separated/divorced<br>4. widowed<br>9. don't know	3 → 3<br>4 → 2<br>9 → -1
Netherlands	Marital status (emarst)	1. never married<br>2. married<br>3. divorced<br>4. widowed	1 →0<br>2 → 1<br>3 → 3<br>4 → 2
Spain	Marital status (Marital_status)	1. single<br>2. married<br>3. widowed<br>4. separated/divorced<br>5. free union<br>6. no answer	1 → 0<br>2 → 1<br>3 → 2<br>4 → 3<br>5 → 0<br>6 → -1
UK	Hhq: marital status (hmstat)	1. single<br>2. married<br>3. divorced or separated<br>4. widowed<br>5. cohabiting	1 → 0<br>2 → 1<br>3 → 3<br>4 → 2<br>5 → 0

SOCIO-ECONOMIC STATUS

STUDY	QUESTION (variable name)	VALUE	EPOSA CODE
	**Education**	(edu)	-1. missing<br>1. Elementary school not completed<br>2. Elementary school completed<br>3. Vocational education or general secondary education<br>4. College or university education
Germany	Highest academic qualification (IV1N010)	1. none (incl. persons having attended general school for at least seven years)<br>2. secondary general school-leaving certificate<br>3. intermediate school-leaving certificate<br>4. Fachhochschule/University entrance qualification<br>5. abitur<br>6. college certificate<br>7. university certificate<br>8. other	1 → 1<br>2 → 2<br>3 → 2<br>4 → 3<br>5 → 3<br>6 → 4<br>7 → 4<br>8 → -1
Italy	Highest educational level (schooltitle)<br><br>Years of education (yschool)	1. none<br>2. less than primary school<br>3. primary school completed<br>4. secondary school completed<br>5. corsi prof completed<br>6. high school completed<br>7. pre-university completed<br>8. university completed<br>9. don’t know<br><br>range: …	1 → 1<br>2 → 1<br>3 → 2<br>4 → 3<br>5 → 3<br>6 → 4<br>7 → 4<br>8 → 4<br>9 → -1

Netherlands	Education level attained<br>(aeducat)	1. elementary not completed<br>2. elementary education<br>3. lower vocational education<br>4. general intermediate education<br>5. intermediate vocational education<br>6. general secondary education<br>7. higher vocational education<br>8. college education<br>9. university education	1 → 1<br>2 → 2<br>3,4,5,6 → 3<br>7,8,9 → 4
Spain	What level of education do you have<br>(education)	1. neither read nor write<br>2. did not finish primary school<br>3. complete primary education<br>4. secondary school/high school<br>5. university<br>9. no answer	1,2 →1<br>3 → 2<br>4 → 3<br>5 → 4
UK	Age (yrs) finished ft education<br>(hendedu)<br><br>Age (yrs) finished ft education (bands) (hendeduc)	range: 11-27<br>(In the UK, an individual; can leave school at 16. Thereafter, they may go to college/ enter a vocational scheme/ stay at school until 18. Thereafter, they may go to University until age 21-24)<br><br>1. ≤14<br>2. ≥15	<15 → 1<br>15-17 → 2<br>18-20 → 3<br>>20 → 4
	**Income**	(income)	-1. missing<br>0. <500 Euro<br>1. ≥500 Euro
Germany	Not available		
Italy	Monthly income (incomep)	1. ≥500 euro<br>2. <500<br>3. refusal<br>9. don't know	1 → 1<br>2 → 0<br>3,9 → -1
Netherlands	Income categories in guilders<br>(einccat)	-2. no answer<br>1. f 1.000 – 1.250	-2 → -1<br>1 → 0

		2. f 1.251 – 1.500<br>3. f 1.501 – 1.750<br>4. f 1.751 – 2.000<br>5. f 2.001 – 2.250<br>6. f 2.251 – 2.500<br>7. f 2.501 – 3.000<br>8. f 3.001 – 3.500<br>9. f 3.501 – 4.000<br>10. f 4.001 – 4.500<br>11. f 4.501 – 5.000<br>12. >f 5.000	2-12 → 1
Spain	What is the total amount of the income (income_home)	1. None<br>2. less than 120 euros<br>3. >=120 to <300<br>4. >=300 to <600<br>5. >=600 to <900<br>6. >=900 to <1200<br>7. >1200<br>9. no answer	1-4 → 0<br>5-7 → 1<br>9 → -1
UK			
	**Socio-economic status**	(SES)	-1. missing<br>1. I large employers and higher managerial occupations, higher professional occupations<br>2. II lower managerial and professional occupations<br>3. III intermediate occupations<br>4. IV small employers and own account workers<br>5. V lower supervisory and technical occupations<br>6. VI semi-routine occupations<br>7. VII routine occupations
Germany	Main occupation during your life (IV1N019)	1. leading position (e.g. manager, director)<br>2. middle leading position (e.g. master, supervisor)<br>3. white-collar worker (administrator, assistant)<br>4. blue-collar worker (e.g. salesman)<br>5. trained operator	1 → 1<br>2 → 2<br>3 → 3<br>4 → 4<br>5 → 5

		6. untrained operator<br>7. self-employed (e.g. craftsman, tradesman)<br>8. freelancer (e.g. doctor, lawyer)<br>9. housewife/househusband<br>99. no answer	6 → 7<br>7 → 4<br>8 → 1<br>9 → 7<br>99 → -1
Italy	Type of work before retirement (WO2)	0 None<br>0.10 Accountant and auditor of financial statements<br>0.11 Chemist<br>0.12 Physical<br>0.13 Geologist, geophysicist, meteorologist, astronomer<br>0.14 Technician, physical and chemical sciences.<br>0.15 Specialist in physical and chemical sciences and assimilated technician<br>0.21 Architect and urban planner<br>0.22 Engineer, civil.<br>0.23 Engineer, electrical and electronic<br>0.24 Engineer, mechanical<br>0.25 Engineer, chemical<br>0.26 Engineer, metallurgical<br>0.29 Engineer, mining<br>0.30 Engineer, work organization, or in areas such as ceramics, glass<br>0.31 Surveyor<br>0.32 Designer<br>0.33 Technician, engineering/civil.<br>0.34 Electrical and electronic engineering.<br>0.35 Technician, mechanics.<br>0.36 Technician, industrial chemistry .<br>0.37 Technician, metallurgy.<br>0.38 Technician, mining.<br>0.39 Technician, industry - methods, time, work.<br>0.41 Pilots, navigators and mechanics of air and sea navigators<br>0.42 Officer and pilot of sea and river.	0 → -1<br>0.1-1.99 → 1<br>2.0-2.99 → 2<br>3.0-3.99 → 3<br>4.0-4.99 → 4<br>5.0-5.99 → 5<br>6.0-8.99 → 6<br>9.0-10.0 → 7

		0.43 Officer, mechanics.<br>0.51 Biologists, zoologists, and specialists.<br>0.52 Bacteriologist, pharmacologist, biochemist, specialist in human and animal pathology<br>0.53 Agronomy.<br>0.54 Technician, biologial, agricultural and medical sciences<br>0.61 Doctor and surgeon.<br>0.62 Assistant, medical.<br>0.63 Dentists.<br>0.64 Assistant, dental.<br>0.65 Veterinary.<br>0.66 Assistant, veterinary.<br>0.67 Pharmacist.<br>0.68 Assistant, pharmacist.<br>0.69 Dieticians and specialists of the problems of nutrition.<br>0.71 Nurse, professional.<br>0.72 Nurse, generic.<br>0.73 Midwive, professional.<br>0.74 Midwive, not graduate .<br>0.75 Optometrist, optician.<br>0.76 Physiotherapist, therapist, masseur.<br>0.77 Technician, medical radiology.<br>0.79 Doctor, orthopaedic/hygienist.<br>0.81 Statisticians.<br>0.82 Mathematician and actuarial science.<br>0.83 Analyst, systems/database<br>0.84 Technician, statistica/mathematic/computer.<br>0.90 Economist and specialist in marketing.<br>1.21 Lawyer, attorney and legal adviser.<br>1.22 Judge<br>1.29 Operator, legal sector/consultant for companies/etc..<br>1.31 Professor, university/higher school.	

		1.32 Teacher, secondary education.<br>1.33 Teacher, primary education.<br>1.34 Teacher, pre-primary education.<br>1.35 Director/inspector/programmer, school.<br>1.41 Minister/member, religion.<br>1.49 Other members of the clergy and healers.<br>1.51 Author and critic.<br>1.59 Author, journalist, writer, such as: editorial secretary, commentator<br>1.61 Sculptor, painter, artist, designer of caricatures, restorer<br>1.62 Decorators window dresser, designer, fashion designer<br>1.63 Photographers, cameramen, reporters.<br>1.71 Composer, musician, singer, conductor, choir, brass band<br>1.72 Choreographer and dancer.<br>1.73 Actor and director.<br>1.74 Manufacturer of cinema, theater, radio and television<br>1.75 Artist, cinema.<br>1.79 Announcer, presenter, animator of the show.<br>1.80 Athlete, sportsman and coache.<br>1.91 Librarian, archivist.<br>1.92 Sociologist, anthropologist, psychologist, geographer, historian<br>1.93 Social worker and cultural entertainment.<br>1.94 Specialist, staff problems/training.<br>1.95 Philologist, translator and interpreter.<br>1.99 Engineer, brevets, advertising campaigns, insurers<br>2.01 Members of the legislative body<br>2.02 Senior official, public administration.<br>2.11 Director-general.<br>2.12 Production director.	

		2.19 Director, sales, research, accounting, staff and transport<br>3.00 Department head in private or public companies.<br>3.10 Intermediate Official in the public sector.<br>3.21 Stenographer, typist, teletype.<br>3.22 Operator, drilling machines.<br>3.31 Assistant/clerk, accounts, accounting machine, bank, post office<br>3.39 Clerk, payroll/contributions.<br>3.41 Labourer, accounting machines and calculators.<br>3.42 Labourer, machines for automatic processing of data<br>3.51 Head station.<br>3.52 Head of the postal services.<br>3.59 Manager/controller, transport services (land, air, etc.)<br>3.60 Heads and train conductors.<br>3.70 Messenger and postman.<br>3.80 Workers telephones and telegraphs.<br>3.91 Clerk, shipping and receiving goods, warehousemen, laundry workers weighing<br>3.92 Clerk, purchasing departments.<br>3.93 Clerk, administrative services.<br>3.94 Clerk, acceptance/travel offices.<br>3.95 Clerk, library and information binders, proofreader drafts<br>4.00 Director, wholesale and retail.<br>4.10 Owners-managers, wholesale and retail trade.<br>4.21 Heads sales.<br>4.22 Worker, supply.<br>4.31 Agents trade-technical, and commercial and technical inspectors.<br>4.32 Touts, agents and traveling salesmen.<br>4.41 Insurance agents, real estate agents and stock exchanges	

		4.42 Seller, business services/advertising space.<br>4.43 Seller, auction; estimator, merchandise.<br>4.51 Salesman, clerk-commercial, demonstrator, mannequin<br>4.52 Peddler/Seller, newspapers/kiosks in general<br>4.90 Other staff of Commerce (pawnshop).<br>5.00 Directors hotels, bars, restaurants, purser.<br>5.10 Owners-managers of restaurants, cafes, hotels.<br>5.20 Director room, intendant, bursar.<br>5.31 Servants, barmen, Sommellerie.<br>5.40 Housekeeper, caretaker of the hotel, Mistress, maid plans, baby sitter<br>5.51 Keeper of buildings.<br>5.52 Maid Service, cleaner in homes.<br>5.60 Officer, washing/ironing/cleaning of linen.<br>5.70 Hairdresser, beautician, manicure.<br>5.81 Fireman.<br>5.82 Policeman, public/private.<br>5.89 Other personnel providing security services.<br>5.91 Guide.<br>5.92 Officer, funeral/embalming<br>5.99 Bookmakers, croupiers, steward.<br>6.00 Director/head, farms.<br>6.11 Farm worker, polyvalent.<br>6.12 Farm worker, skilled<br>6.2 Employees.<br>6.21 Roundsman, many tasks.<br>6.22 Officer, field cropping or vegetables.<br>6.23 Officer, growing trees and shrubs.<br>6.24 Officer, drystock breeding.<br>6.25 Officer, dairy breeding.<br>6.26 Officer, poultry rearing.<br>6.27 Officer, vivarium and gardens.<br>6.28 Conductor of agricultural machinery.<br>6.29 Beekeeper, breeding silkworms, harvesting	

		resins, irrigator, gardener<br>6.31 Woodman.<br>6.32 Workers forest except woodcutters.<br>6.41 Fisherman.<br>6.49 Farmer, fish/oyster; hanter, fur/seals/whale<br>7.00 Department Heads, Heads workers.<br>7.11 Miner and quarrier.<br>7.12 Workers of the initial processing of the extracted material<br>7.13 Driller, cemented, acidification of wells, reps probes<br>7.21 Steel furnaces conductor: blast furnaces, electric furnaces<br>7.22 Lemina, both ferrous and non ferrous materials.<br>7.23 Secondary smelting/reheating furnaces conductor.<br>7.24 Metal Colatore: pouring molten metal into molds.<br>7.25 Makers of molds and cores for casting metals.<br>7.26 Workers at theheat treatment of metals: temperature<br>7.27 Conductor, dies and presses.<br>7.28 Workers at electrogalvanic procedure for the protective coating<br>7.29 Additional production workers and processing of metals: eg. Cleaners<br>7.31 Workers to chemical treatment of wood.<br>7.32 Workers preparing the wood sawyers, collators, breeders<br>7.33 Workers preparing pulp for making paper.<br>7.34 Workers who process the dough into paper.<br>7.41 Tritatori, mixers, crushers for chemicals.<br>7.42 Conductors furnaces & equipment for heat treatment of chemicals substances<br>7.43 Conductors filtering apparatus for separating oil from sediment<br>7.44 Conductors and equipment used for the	

		production of reaction<br>7.45 Workers refining of oil.<br>7.49 Conductors of furnaces and equipment for the chemical processing of synthetic fibers<br>7.51 Textile fiber preparer: sorting, washing, blending, battitu<br>7.52 Yarn spinners, twisters, winders coils.<br>7.53 Workers maintenance and adjustment of frames.<br>7.54 Orditori, weaver of fabrics, upholstery, carpets.<br>7.55 Weavers knitwear and hosiery.<br>7.58 Washers, bleaching, dyeing of yarn and fabrics.<br>7.59 Workmen to finishing of textiles: washers in seltro, workers at<br>7.61 Cernitori, tanners, dyers, depilatories, dresser of skins<br>7.62 Cernitori, tanners, dyers, depilatories, dresser of furs<br>7.71 Millers and production staff of rice and spices.<br>7.72 Employees in production and refining of sugar.<br>7.73 Butchers and meat processors, including pork.<br>7.74 Workers for the cultivation and preservation of preserves.<br>7.75 Workers treatment of milk and its derivatives.<br>7.76 Bakers and confectioners.<br>7.77 Workers in the preparation of tea, coffee and cocoa<br>7.78 Workers of the preparation of malt, wine, vinegar, fruit juices<br>7.79 Workers in the production of vegetable oil and canned fish<br>7.81 Packer of cigars<br>7.83 Packer cigarettes<br>7.89 Mixers tobacco, preparatory pressed for pipe tobacco<br>7.91 Complete tailors, cutters and packers clothes	

		tailor-cutter<br>7.92 Cutters, sewers dresses in fur.<br>7.93 Modiste boxes of hats.<br>7.94 Designers of models, dressed in leather cutter.<br>7.95 Sewers and embroiderers.<br>7.96 Upholsterers and packers of mattresses.<br>7.99 Packer of tents and tarpaulins, editors of umbrellas<br>8.01 Clothes and shoes repair entirely to order.<br>8.02 Shoe factory worker.<br>8.03 Worker of leather: cutting, sewing, riding<br>8.11 Carpenter, made of furniture.<br>8.12 Conductor machines for sawing, shaping, carving wood<br>8.19 Wooden beaters, rivestitori, restorers, inlayers, sculptors<br>8.20 Cutters, finishers, sculptors, engravers, cleaning of stones.<br>8.31 Smith, blacksmith and forger.<br>8.32 Tools-fitter tool, repair of metal models<br>8.33 Regulators-conductors of machine tools. milling machines, lathes, grinders etc..<br>8.34 Conductors of machine tools.<br>8.35 Polishers, grinders, wire presses, shearing machines, bending<br>8.39 Gunsmith, editor of locks.<br>8.41 Manufacture, repair, install machine tools and other mechanical devices<br>8.42 Watchmakers and mechanics of precision optical instruments, prostheses ortopedich<br>8.43 Mechanics of motor vehicles.<br>8.44 Mechanics of aircraft engines.<br>8.48 Fitters, steam fitters, diesel engines, turbines<br>8.51 Adjusters of electrical and electromechanical<br>8.52 Adjusters electronic devices.	

		8.53 Assemblers of electrical and electronic equipment and components manufacture.<br>8.54 Receptors repair of radio and television.<br>8.55 Electricians in domestic installations, ships, aircraft, and maintenance<br>8.56 Fitters installation of telephone and telegraph.<br>8.57 Fitters of power lines.<br>8.58 Electricians and electrical workers to quality control during the prod<br>8.61 Operators of radio and television broadcasting stations<br>8.62 Operators of equipment for sound and projection of cinema<br>8.71 Plumbers and pipe fitters installers.<br>8.72 Welders and cutters with the oxyhydrogen blowpipe<br>8.73 Fitters repair of sheet metal parts. body, boilers etc.<br>8.74 Fitters carpentry and metal structures.<br>8.80 Jewelers and goldsmiths.<br>8.91 Blowers, grinders, cutters, cernitori and cleaning of glassware<br>8.92 Potters, molders, colatori, pressers of clay.<br>8.93 Conductors furnaces in glass and ceramic kilns, pottery, bricks<br>8.94 Engravers vitrified products.<br>8.95 Painters on glass and ceramics.<br>8.99 Preparation of meals of clay, abrasive paste, fiber glass<br>9.01 Workers of the manufacture of rubber and plastic: grinders<br>9.02 Makers and vulcanization of tires.<br>9.10 Packers on paper and cardboard, assembled by hand and machine<br>9.21 Typesetting. composers and printers in general,	

		composers<br>9.22 Conductors printing machines, rotary presses, offset lithographic<br>9.23 Stereotypical molds to make the typesetting<br>9.24 Engravers plates for printing.<br>9.25 Engravers.<br>9.26 Bookbinder, embossing cover.<br>9.27 Print, development and enlargement in the darkroom films.<br>9.29 Printmakers fabric and screen printing.<br>9.31 Painters, coatings, lacquers constructions.<br>9.39 Painters roller and brush for decoration and protection, wood<br>9.41 Luthiers, manufacturers and tuners for musical instruments.<br>9.42 Handmade baskets, wicker, rattan, brushes, brooms, furniture<br>9.43 Producers of manufactured concrete and asbestos cement for construction<br>9.49 Stuffed animals, and manufacture of linoleum, dolls and games<br>9.51 Mason employees in general, builder of brick walls<br>9.52 Workers of reinforced concrete, owners of cages for reinforced concrete.<br>9.53 Copritori of roofs slate, tiles, plastic, asphalt<br>9.54 Wooden Carpenters, joiners, parquet floor in buildings, ships and aircraft.<br>9.55 Plasterers, Plasterer.<br>9.56 Applicators insulation and soundproofing material.<br>9.57 Glaziers, tilers, glaziers-decorators, glaziers automobile.<br>9.59 Mason track on his own, mason maintenance, paper and cloth upholstery	

		9.61 Conductors and employees to power for the production of electricity.<br>9.69 Conductors fixed machinery: air/gas compressors, stoker boilers<br>9.71 Loading and unloading of ships and land vehicles, warehouse worker<br>9.72 Fitters, repairers, equipped with apparatus for elevation, cables on<br>9.73 Conductors of cranes and mobile towers, plant elevation.<br>9.74 Conductors diggers, of bulldozers, dredges, bulldozers, bitumier<br>9.79 Conductors truck, tipper truck, bus service in the mines<br>9.81 Heads of sailors cleaners, qualified rating.<br>9.82 Mariner engine room.<br>9.83 Lead locomotives, suburban trains and mining.<br>9.84 Head freight train, engaged in trade, coupled switcher<br>9.85 Lead trams, taxis, buses, trucks, trucks, motorcycles, motor tricycles<br>9.86 Conductors of animal-drawn vehicles.<br>9.89 Traffic Controllers ports, locks, lighthouse keepers<br>9.99 Laborers in general<br>10 Housewife	
Netherlands	Which occupation did you have during the longest period in your life? (bjoblo2, bljclass)<br><br>Longest job coded into bljclass using the occupational class SBC92.	11. elementary occupations<br>21. lower non-specialized occupations<br>22. low teaching and instruction occupation<br>24. lower agricultural occupations<br>25. lower natural science occupations<br>26. lower technical occupations<br>28. lower transport occupations<br>29. lower (para)medical occupations<br>31. lower administrative & commercial occupations	11 → 7<br>21 → 7<br>22 to 37 → 5<br>44 to 57 → 3<br>62 to 78 → 2<br>82 to 98 → 1<br>999 to 9999 → -1

		33. lower security occupations<br>37. lower service & care occupations<br>44. medium agricultural occupations<br>45. medium natural science occupations<br>46. medium technical occupations<br>48. medium transport occupations<br>49. medium (para)medical occupations<br>51. medium administrative & commercial occupations<br>53. medium juridical & security occupations<br>55. medium linguistic & cultural occupations<br>56. medium social occupations<br>57. medium service & care occupations<br>62. higher pedagogical occupations<br>64. higher agricultural occupations<br>65. higher natural science occupations<br>66. higher technical occupations<br>68. higher transport occupations<br>69. higher (para)medical occupations<br>71. higher administrative & commercial occupations<br>73. higher juridica & security occupations<br>75. higher linguistic & cultural occupations<br>76. higher social occupations<br>78. higher managers<br>82. scientific pedagogical occupations<br>85. scientific natural science occupations<br>86. scientific technical occupations<br>89. scientific (para)medical occupations<br>91. scientific administrative & economic occupations<br>93. scientific juridical & policy occupations<br>96. scientific social occupations<br>98. scientific managers<br>999. missing<br>9999. not available	
Spain	What has been your main occupation? Occupation was	1. I Large employers and graduated of 2 or 3 cycles of university	1 → 1<br>2 → 2

	classified using the National Classification of Occupations (CON-1989) but with an extra category for housewives. (Salvany et al, Propuesta de un indicador de la clase social basado en la occupación. Gac Sanit 1989; 3(10):320-326) (occupation)	2. II Young employees and graduates of 1 cycle of university<br>3. IIIa administrative and security<br>4. IIIb own account workers<br>5. IVa skilled manual workers<br>6. IVb semi-skilled manual workers<br>7. V unskilled<br>8. housewifes	3 → 3<br>4 → 4<br>5 → 5<br>6 → 6<br>7 → 7<br>8 → 7
UK	Current NS-SSEC (National Statistics Socioeconomic Classification) (nssec)	1. I<br>2. I<br>3. II<br>4. III<br>5. IV<br>6. V<br>7. VI<br>8. VII	1 → 1<br>2 → 1<br>3 → 2<br>4 → 3<br>5 → 4<br>6 → 5<br>7 → 6<br>8 → 7

MEDICAL STATUS

STUDY	QUESTION (variable name)	VALUE	EPOSA CODE
	**Cardiovascular diseases**	Ever had a cardiovascular disease (cvd)	-1. missing<br>0. no<br>1. yes
Germany	Has a doctor ever told you that you have or had a myocardial infarction? (IV1N104)<br><br>Has a doctor ever told you that you have or had a heart failure? (IV1N106)	1. no<br>2. yes, still causes impairment<br>3. yes, no impairment anymore<br><br>dito	1 → 0<br>2 → 1<br>3 → 1<br>If yes on either one of the questions → 1<br>If no on all questions → 0<br>If missing on any of the questions and no on the other questions → -1
Italy	Chronic heart disease:<br>- angina (angina)<br>- myocardial infarction (infarction)<br>- heart failure (heart_failure)	1. definite<br>2. possible<br>3. no	After discussion with Sabina, "possible" was defined as no:<br>If yes on either one of the questions → 1<br>If no or possible on all questions → 0<br>If missing on any of the questions and no/possible on the other questions → -1
Netherlands	Do you have a heart disease or have you had a myocardial infarction? (ehart01)	-5. no interview<br>-1. not available, asked<br>0. no, never<br>1. no, yes previous cycle<br>2. yes, no on previous cycle<br>3. yes, yes on previous cycle	-5 → -1<br>-1 →-1<br>0 → 0<br>1 → 0<br>2 → 1<br>3 → 1
Spain	Heart disease (Heart)	1. yes<br>2. no<br>2000. missing	1 → 1<br>2 →0<br>2000 → -1
UK	Hhq: gp/doctor defined heart attack (hdocha)<br>Hhq: gp/doctor defined angina (hdocang)	0. no<br>1. yes	0 → 0<br>1 → 1<br>If yes on either one of the questions → 1<br>If no on all questions → 0<br>If missing on any of the questions and no on the other questions → -1

	**Arterial diseases**	Ever had an arterial disease (artd)	-1. missing<br>0. no<br>1. yes
Germany	Not available		
Italy	arterial diseases (peripheral artery desease) (arterial)	1. definite<br>2. possible<br>3. no	Possible defined as no<br>If yes on either one of the questions → 1<br>If no on/possible all questions → 0<br>If missing on any of the questions and no/possible on the other questions → -1
Netherlands	Do you have diseases or abnormalities of the arteries or blood-vessels in your belly or legs? (eartvei1)	-5. no interview<br>-1. not available, asked<br>0. no, never<br>1. no, yes previous cycle<br>2. yes, no on previous cycle<br>3. yes, yes on previous cycle	-5 → -1<br>-1 →-1<br>0 → 0<br>1 →<br>2 → 1<br>3 → 1
Spain	Circulation disorders (Circulation)<br><br><br><br>Have you visited the doctor in the last year for that problem? (Circulation_d)	1. yes<br>2. no<br>2000. missing<br><br>dito	1 → 1<br>2 → 0<br>2000 → -1
UK	Not available		
	**Diabetes Mellitus**	Ever had diabetes mellitus (diab)	-1. missing<br>0. no<br>1. yes
Germany	Has a doctor ever told you that you have or had diabetes? (IV1N118)	1. no<br>2. yes, still causes impairment<br>3. yes, no impairment anymore	1 → 0<br>2 → 1<br>3 → 1
Italy	Diabetes (diabetes)	1. definite<br>2. possible<br>3. no	1 → 1<br>2,3 → 0
Netherlands	Do you have diabetes? (ediabe01)	-5. no interview<br>-1. not available, asked<br>0. no, never<br>1. no, yes previous cycle	-5 → -1<br>-1 →-1<br>0 → 0<br>1 →

		2. yes, no on previous cycle<br>3. yes, yes on previous cycle	2 → 1<br>3 → 1
Spain	Diabetes (Diabetes)<br><br><br><br>Have you visited the doctor in the last year for that problem? (Diabetes_d)	1. yes<br>2. no<br>9. missing<br><br>dito	1 → 1<br>2 → 0<br>9 → -1
UK	Ever been told had diabetes (hdiab)	0. no<br>1. yes<br>9. don’t know	0 → 0<br>1 → 1<br>9 → -2
	**Stroke**	Ever had a stroke (cva)	-1. missing<br>0. no<br>1. yes
Germany	Has a doctor ever told you that you have or had a stroke? (IV1N108)	1. no<br>2. yes, still causes impairment<br>3. yes, no impairment anymore	1 → 0<br>2 → 1<br>3 → 1
Italy	Stroke (stroke)	1. yes<br>2. possible<br>3. no	1 → 1<br>2 → 0<br>3 → 0
Netherlands	Did you ever have a stroke or attack? (ecva01)	-5. no interview<br>-1. not available, asked<br>0. no, never<br>1. no, yes previous cycle<br>2. yes, no on previous cycle<br>3. yes, yes on previous cycle	-5 → -1<br>-1 →-1<br>0 → 0<br>1 →<br>2 → 1<br>3 → 1
Spain	Strokes (Strokes)	1. yes<br>2. no<br>20000. missing	1 → 1<br>2 → 0<br>20000 → -1
UK	Ever been told had stroke/tia (hstroke)	0. no<br>1. yes<br>9. don’t know	0 → 0<br>1 → 1<br>9 → -1
	**Malignant neoplasms (cancer)**	Ever had cancer (cancer)	-1. missing<br>0. no<br>1. yes

Germany	Has a doctor ever told you that you have or had cancer? (IV1N122)<br><br>What sort of cancer? Please name (IV1N123)	1. no<br>2. yes, still causes impairment<br>3. yes, no impairment anymore<br><br>String	1 → 0<br>2 → 1<br>3 → 1
Italy	Cancer (cancer)	1. definite<br>2. possible<br>3. no	1 → 1<br>2 → 0<br>3 → 0
Netherlands	Do you have a tumour or cancer or have you ever had it? (ecancer1)	-5. no interview<br>-1. not available, asked<br>0. no, never<br>1. no, yes previous cycle<br>2. yes, no on previous cycle<br>3. yes, yes on previous cycle	-5 → -1<br>-1 →-1<br>0 → 0<br>1 →<br>2 → 1<br>3 → 1
Spain	Neoplastic process (Neoplastic)	1. yes<br>2. no<br>20000. missing	1 → 1<br>2 → 0<br>20000 → -1
UK	Not available		
	**Chronic non-specific lung disease**	Ever had a chronic non-specific lung disease (cnsld)	-1. missing<br>0. no<br>1. yes
Germany	Has a doctor ever told you that you have:<br>- asthma (IV1N089)<br>- pulmonary emphysema (IV1N097)<br>- chronical bronchitis (IV1N099)<br>- COPD (IV1N101)	0. no<br>1. yes	If (IV1N089=1 or IV1N097=1 or IV1N099=1 or IV1N101=1 or IV1N092=1 or IV1N098=1 or IV1N100=1 or IV1N102=1) → 1<br><br>If (IV1N089=0 and IV1N097=0 and IV1N099=0 and IV1N101=0 and IV1N092=0 and IV1N098=0 and IV1N100=0 and IV1N102=0) → 0
Italy	chronic non-specific lung diseases (chronic)	1. definite<br>2. possible<br>3. no	
Netherlands	Do you have CARA (asthma, chronical bronchitis or pulmonary-emphysema)? (ecara01)	-5. no interview<br>-1. not available, asked<br>0. no, never	-5,-1 → -1<br>0,1 → 0<br>2,3 → 1

		1. no, yes previous cycle<br>2. yes, no on previous cycle<br>3. yes, yes on previous cycle	
Spain	Respiratory diseases (Respiratory)	1. yes<br>2. no<br>20000. missing	1 → 1<br>2 → 0<br>20000 → -1
UK	hhq: seen doctor in last year for chest (hdocch)	0. no<br>1. yes<br>9. don’t know	0 → 0<br>1 → 1<br>9 → -1
	**Medication use**	(meduse)	-1. missing<br>0. no<br>1. yes
Germany	Scanning of barcodes of medication. Information available on medication use. (No variables generated yet)		
Italy	Medication use (medication)	1. yes<br>2. no	1 → 1<br>2 → 0
Netherlands	During the past two weeks, did you take any medicine prescribed by a doctor? (emvar13)	-5. no interview<br>-1. not available, asked<br>1. no<br>2. yes	-5,-1 → -1<br>1 → 0<br>2 → 1
Spain	Medication: yes/no (medications_yes_no)	0. no<br>1. yes	0 → 0<br>1 → 1
UK	Drugs categories:<br>-cardiovascular system (hdrugtc)<br>-respiratory sytem (hdrugtr)<br>-gastro-intestinal system (hdrugtg)<br>-endocrine (hdrugth)<br>-central nervous system (hdrugtn)<br>-malignant disese & immunosuppression (hdrugtm)<br>-nutrition & blood (hdrugts)<br>-musculoskeletal & joint disease (hdrugtj)<br>-eye (hdrugte)	0. no<br>1. yes<br>9. don’t know	If yes on any of the drugs listed → 1<br>If no on all of the drugs listed → 0<br>If missing on any of the drugs and no on all of the other drugs listed → -1

	-ear (hdrugta)<br>-nose (hdrugto)<br>-skin(hdrugtd)<br>-miscellaneous (hdrugtq)<br>-genito-urinary tract (hdrugtu)		
	**Number of medications**	(nmed)	-1. missing<br>Range 0-8<br>(8=8 or more)
Germany	Scanning of barcodes of medication. Information available on number of medication (No variables generated yet)		
Italy	number of medication (nmedication)		
Netherlands	The name, dose and frequency of a maximum of 8 medications were copied from the containers , the total number of medication was counted (em#med)	-2. not available<br>range 0-8	-2 → -1<br>0-8 → copy
Spain	Total number of medications (Medications_number)	range: 0-highest	0-8 → copy<br>9-highest → 8
UK	Sum of the following variables:<br>thiazides & related diuretics (hdrugc1)<br>frusemide (hdrugc2)<br>spironolactone (hdrugc3)<br>other diuretics (hdrugc4)<br>potassium supplements (hdrugc5)<br>beta-blockers only (hdrugc6)<br>digoxin (hdrugc7)<br>amiodarone (hdrugc8)<br>other anti-arrhythmic drugs (hdrugc9)<br>ace inhibitors (hdrugc10)<br>angiotensin-ii receptor antagonists (hdrugc11)<br>calcium-channel blockers (hdrugc12)	0. no<br>1. yes<br>9. don't know	If 9 on all variables → -1.<br>Sum all variables.<br>0-8 → copy<br>9-highest → 8

	nitrates (hdrugc13)<br>nicorandil (hdrugc14)<br>alpha-adrenoreceptor blocking drugs (hdrugc15)<br>warfarin (hdrugc16)<br>aspirin (hdrugc17)<br>clopidogrel (hdrugc18)<br>dipyridamole (hdrugc19)<br>peripheral & cerebral vasodilators (hdrugc20)<br>statins (hdrugc21)<br>fibrates (hdrugc22)<br>other moxonidine (hdrugc23)<br>inhaled steroids (hdrugr1)<br>inhaled selective (2-adrenoreceptor stimulant) (hdrugr2)<br>inhaled antimuscarinic bronchodilators (hdrugr3)<br>aminophllyine / theophylline (hdrugr4)<br>antihistamines (hdrugr5)<br>other singulair (hdrugr6)<br>h2-receptor antagonists (hdrugg1)<br>proton pump inhibitors (hdrugg2)<br>antacids (hdrugg3)<br>cisapride (hdrugg4)<br>anti-emetics (hdrugg5)<br>ispaghula husk (hdrugg6)<br>bulk-forming laxatives (excluding ispaghula) (hdrugg7)<br>senna (hdrugg8)<br>stimulant laxatives (excluding senna) (hdrugg9)<br>liquid paraffin (hdrugg10)<br>softener laxatives (excluding liquid paraffin) (hdrugg11)<br>lactulose (hdrugg12)		

	magnesium salts (hdrugg13)<br>osmotic laxatives (excluding lactulose, etc) (hdrugg14)<br>anti-diarrhoeal drugs (hdrugg15)<br>anti-spasmodics (hdrugg16)<br>aminosalicylates (hdrugg17)<br>other misoprostol (in arthrotec & napratec) (hdrugg18)<br>insulin (hdrugh1)<br>sulphonylureas (hdrugh2)<br>biguanides (hdrugh3)<br>other oral diabetes drugs (hdrugh4)<br>thyroxine (hdrugh5)<br>carbimazole and other hyperthyroidism drugs (hdrugh6)<br>female hormone replacement therapy (hdrugh7)<br>male sex hormones (hdrugh8)<br>growth hormone (hdrugh9)<br>oral steroids (hdrugh10)<br>other desmospray (hdrugh11)<br>benzodiazepines (hdrugn1)<br>hypnotics and anxiolytics (excluding benzodiazepine) (hdrugn2)<br>anti-psychotics (hdrugn3)<br>lithium (hdrugn4)<br>ssris (selective serotonin reuptake inhibitor) (hdrugn5)<br>tcas (tricyclic antidepressants) (hdrugn6)<br>antidepressants (excluding ssris and tcas) (hdrugn7)<br>paracetamol (hdrugn8)<br>codeine containing drugs (hdrugn9)<br>opioid containing drugs (hdrugn10)<br>anticonvulsant drugs (hdrugn11)		

	migraine drugs (hdrugn12)<br>l-dopa containing drugs (hdrugn13)<br>other parkinsons disease drugs (hdrugn14)<br>drugs for vestibular disorders (hdrugn15)<br>other lioresal - baclofen (hdrugn16)<br>tamoxifen (hdrugm1)<br>prostate cancer drugs (hdrugm2)<br>other (hdrugm3)<br>vitamin a (hdrugs1)<br>vitamin b1 (thiamine) (hdrugs2)<br>vitamin b2 (riboflavin) (hdrugs3)<br>vitamin b6 (pyridoxine) (hdrugs4)<br>nicotinamide (hdrugs5)<br>vitamin b12 (hdrugs6)<br>vitamin c (hdrugs7)<br>vitamin d only (hdrugs8)<br>vitamin e (hdrugs9)<br>vitamin k (hdrugs10)<br>multivitamins (hdrugs11)<br>calcium (hdrugs12)<br>calcium & vitamin d (hdrugs13)<br>iron supplements (hdrugs14)<br>folic acid (hdrugs15)<br>cod liver oil (hdrugs16)<br>fish oils (hdrugs17)<br>evening primrose oil (hdrugs18)<br>fortisip, ensure and other supplement drinks (hdrugs19)<br>other (hdrugs20)<br>nsaids (hdrugj1)<br>cox-2 specific inhibitors (hdrugj2)<br>bisphosphonates (hdrugj3)<br>raloxifene (hdrugj4)<br>allopurinol and other gout drugs (hdrugj5)<br>rheumatic disease modifying drugs (hdrugj6)		

	quinine (hdrugj7)<br>glucosamine sulphate (hdrugj8)<br>chondroitin sulphate (hdrugj9)<br>other (hdrugj10)<br>beta blocker eye drops (hdruge1)<br>miotic eye drops (hdruge2)<br>sympathomimetic eye drops (hdruge3)<br>carbonic anhydrase inhibitors (hdruge4)<br>prostaglandin analogues (hdruge5)<br>tear deficiency eye drops (hdruge6)<br>anti-inflammatory preparations (hdruge7)<br>other chloramphenicol eye ointment (hdruge8)<br>ear drops (hdruga1)<br>other (hdruga2)<br>steroid nasal sprays (hdrugo1)<br>other tramazoline in dexa-rhinaspray duo (hdrugo2)<br>topical steroids (hdrugd1)<br>emollients (hdrugd2)<br>anti-fungal agents (hdrugd3)<br>other 10% coal tar solution with betnovate (hdrugd4)<br>antibiotics (hdrugq1)<br>other daktarin oral gel (hdrugq2)<br>finasteride (hdrugu1)<br>drugs for urinary incontinence (hdrugu2)<br>sildenafil (hdrugu3)<br>drugs for impotence (hdrugu4)<br>other (hdrugu5)		
	**Pain/anti-inflammatory medication**	(painmed)	-1. missing<br>0. no<br>1. yes
Germany	Scanning of barcodes of medication. Information available on pain medication (No variables generated yet)		

Italy	Drugs for joint pain/difficulty of movement (AR36)<br><br><br>Anti-inflamatory/pain medication (medicpain)	1. yes<br>2. no<br>9. don't know<br><br>1. yes<br>2. no	1 →1<br>2 →0<br>9 →-1
Netherlands	ATC-codes were assigned to each drug (ematc1–ematc8)	N01xxxx (anesthetics)<br>N02xxxx (analgetics)<br>M01Axxx (prostaglandines)<br>L04Axxx (anti-inflammatory)	If (ematc1='N01xxxx' or ematc1='N02xxxx' or ematc1='M01xxxx' or ematc1='L04Axxx') painmed=1.<br>Dito for medication 2-8.<br>Else painmed=0.<br>If (sysmis(emvar13)) painmed=-1.
Spain	Medicaments for Osteoarthritis (maximum of 4) in ATC-codes (Note that these medications may be pain medication or anti-inflammatory medication) (OA_med1, OA_med2, OA_med3, OA_med4)	N01xxxx (anesthetics)<br>N02xxxx (analgetics)<br>M01Axxx (prostaglandines)<br>L04Axxx (anti-inflammatory)<br>20000. missing	If (OA_med1='N01' or OA_med1='N02' or OA_med1='M01' or OA_med1='L04A') painmed=1.<br>If (OA_med2='N01' or OA_med2='N02' or OA_med2='M01' or OA_med2='L04A') painmed=1.<br>If (OA_med3='N01' or OA_med3='N02' or OA_med3='M01' or OA_med3='L04A') painmed=1.<br>If (OA_med4='N01' or OA_med4='N02' or OA_med4='M01' or OA_med4='L04A') painmed=1.
UK	Paracetamol (hdrugn8)<br>Codeine containing drugs (hdrugn9)<br>Opioid containing drugs (hdrugn10)	1. yes<br>2. no<br>9. don't know	If (hdrugn8=1 or hdrugn9=1 or hdrugn10=1) → 1<br>If (hdrugn8=0 & hdrugn9=0 & hdrugn10=0) → 0<br>If (hdrugn8=9 & hdrugn9=9 & hdrugn10=0) → -1<br>If (hdrugn8=0 & hdrugn9=0 & hdrugn10=9) → -1<br>If (hdrugn8=0 & hdrugn9=9 & hdrugn10=0) → -1<br>If (hdrugn8=9 & hdrugn9=0 & hdrugn10=0) → -1<br>If (hdrugn8=9 & hdrugn9=0 & hdrugn10=9) → -1<br>If (hdrugn8=0 & hdrugn9=9 & hdrugn10=9) → -1

COGNITIVE AND PSYCHOLOGICAL FUNCTIONING

STUDY	QUESTION (variable name)	VALUE	EPOSA CODE
	**Cognitive functioning**	(cognition)	-1. missing<br>0. good cognitive functioning<br>1. poor cognitive functioning
Germany	Mini-Mental State Examination (no total score generated yet)		MMSE>23 → 0<br>MMSE≤23 → 1
Italy	Mini-mental state examination (MMT)	range: 0-30	MMSE>23 → 0<br>MMSE≤23 → 1
Netherlands	Mini-mental state examination (emmsesc)	-2. too many missings<br>range: 0-30	-2 → -1<br>MMSE>23 → 0<br>MMSE≤23 → 1
Spain	Total score of errors in the test of Pfeiffer (Pfeiffer_errors)	range: 0-11	Pfeiffer_errors<3 → 0<br>Pfeiffer_errors≥3 → 1
UK	Strawbridge frailty questionnaire: frail on Strawbridge cognitive domain (fqscog)	0. no<br>1. yes	0 → 0<br>1 → 1
	**Depressive symptoms**	(depression)	-1. missing<br>0. no depressive symptoms<br>1. depressive symptoms
Germany	HADS (no total score generated yet)	Range: 0-21<br>No case: 0-7<br>Possible case: 8-10<br>Probable case: 11-21	0 -7 → 0<br>≥8 → 1
Italy	Geriatric Depression Scale (GDS)	0-10: no depression<br>11-20: mild depression<br>21-30: severe depression	0-10 → 0<br>>10 → 1
Netherlands	CES-D (ecesdint)	-2. no valid data<br>range 0-60	-2 → -1<br>0 -15 → 0<br>≥16 → 1
Spain	CES-D (Depression_n)	range 0-60	0 -15 → 0<br>≥16 → 1
UK	HAD-D (hhadd)	range: 0-21	0 -7 → 0

		No case: 0-7<br>Possible case: 8-10<br>Probable case: 11-21	≥8 → 1

ANTHROPOMETRY/BODY COMPOSISTION

STUDY	QUESTION (variable name)	VALUE	EPOSA CODE
	**Body height**	(height)	-1. missing<br>range: 0-highest (cm)
Germany	Measured height in meters, centimetres (IV2N534)	range: 0-highest	copy
Italy	Body height in cm (bodyheight)	range: 0.0-highest	copy
Netherlands	Measured height in centimetres (emed150)	-2. not available<br>-1. no (valid) data<br>range 0-highest	copy
Spain	Height (cm) (height_c)	range:0-highest<br>20000. missing	copy
UK	Height (cm) (aht)	range: 0-highest	copy
	**Body weight**	(weight)	-1. missing<br>range: 0-highest (kg)
Germany	Measured weight in kilograms (IV2N529)	range: 0-highest	copy
Italy	Body weight in cm (bodyweight)	range: 0.0-highest	copy
Netherlands	Measured weight in kilograms (emed153)	-2. not available<br>-1. no (valid) data<br>range 0-highest	-2,-1 → -1<br>copy
Spain	Self-reported Weight (kg) (Weightpen)	range: 0-highest	copy
UK	Weight (kg) (awt)	range: 0-highest	copy
	**Body mass index**	(BMI)	-1. missing<br>$weight/(height/100)^2$
	**Waist circumference**	(waist)	-1. missing<br>range: 0-highest (kg)
Germany	Not available<br>Skin thickness at three different positions available as proxy		-1

Italy	Waist circumference, cm (waistprova)	range: 0-highest	copy
Netherlands	Measured waist in cm, two measurements (emed156, emed157)	-2. not available<br>-1. no (valid) data<br>range 0-highest	-2,-1 → -1<br>copy
Spain	Not available		-1
UK	Waist circumference (cm) (await)	range: 0-highest	copy

LIFE STYLE

STUDY	QUESTION (variable name)	VALUE	EPOSA CODE
	**Alcohol use**	(alcohol)	-1. missing<br>0. no<br>1. yes
Germany	How often do you drink alcoholic beverages on average? (IV2N654)	1. daily<br>2. several times during the week<br>3. several times during the month<br>4. less than once a month<br>5. never	1-4 → 1<br>5 → 0
Italy	In the last year:<br>- wine (AL1)<br>- beer (AL3)<br>- liquor (AL5)	1. yes<br>2. no<br>3. abstemious<br>4. don't know	If yes on any of the items → 1<br>If no on all items → 0<br>If abstemious on all items → 0<br>If don't know on all items → -1
Netherlands	Do you drink alcohol? (emalcohu)	-5. not available, interview terminated<br>-1. not available, asked<br>1. no<br>2. yes	1 → 0<br>2 → 1<br>-5,-1 → -1
Spain	Do you drink alcohol regularly (not just at parties or celebrations)? (alcohol_fr)	1. yes<br>2. no<br>9. no answer	1 → 1<br>2 → 0<br>9 → -1
UK	Ever drink alcohol? (hdrink)	0. no<br>1. yes<br>9. dk	0 → 0<br>1 → 1<br>9 →-1
	**Alcohol use per week in categories**	(alccat)	-1. missing<br>0. non-drinker<br>1. very low (0/<1 M&F)<br>2. low (1-10M, 1-7F)<br>3. moderate (11-21M, 8-14F)<br>4. fairly high (22-35M, 15-21 F)<br>5. high (>35M, >21 F)
Germany	Have you ever drunk alcohol in your life?	1. yes	All → -1

	(IV2N651)<br><br>How often do you drink alcoholic beverages on average? (IV2N654)<br><br>How much beer, wine and liquor have you had last weekend (i.e. Saturday AND Sunday)?<br>beer (IV2N655a)<br>lite ale (IV2N655b)<br>must (IV2N655c)<br>wine/champagne (IV2N655d)<br>liquor (IV2N655e)<br><br>How much beer, wine and liquor have you had the last working day? (IV2N656)<br>beer (IV2N656a)<br>lite ale (IV2N656b)<br>must (IV2N656c)<br>wine/champagne (IV2N656d)<br>Liquor (IV2N656e)	2. no<br><br>1. daily<br>2. several times during the week<br>3. several times during the month<br>4. less than once a month<br>5. never<br><br>rounded to 0,5 l<br>rounded to 0,5 l<br>rounded to 0,5 l<br>rounded to 0,25 l<br>Number of shots (2 cl)<br><br>1. monday<br>2. tuesday<br>3. wednesday<br>4. thursday<br>5. friday	
Italy	In the last year:<br>- wine (AL1)<br>- beer in the last month (AL3)<br>- liquor (AL5)<br><br>Liters of … on average in the last month<br>- wine (AL2)<br>- beer (AL41)<br><br>Number of liquor glasses or correct coffee past month (AL61, AL62)	1. yes<br>2. no<br>3. abstemious<br>4. don't know<br><br>range: 0-highest<br><br><br><br>range: 0-highest	Glasses of wine=AL2/4/0.15<br>Glasses of beer=AL41/4/0.25<br>Glasses of liquor=AL61/4. +<br>Glasses of alcohol per week<br><br>Men<br>0 → 0<br><1 → 1<br>1-10 → 2<br>11-21 → 3<br>22-35 → 4

			>35 → 5<br>Women<br>0 → 0<br><1 → 1<br>1-7 → 2<br>8-14 → 3<br>15-21 → 4<br>>21 → 5
Netherlands	Do you drink alcohol? (emalcohu)<br><br><br><br><br>Which of the below mentioned possibilities represents your alcohol use for the past year the closest? (emalcohd)<br><br><br><br><br><br><br><br>When you were drinking alcohol, how many glasses did you usually drink each time? (emalcohn)	-5. not available, interview terminated<br>-1. not available, asked<br>1. no<br>2. yes<br><br>-2. not available<br>1. daily<br>2. 5-6 days a week<br>3. 3-4 days a week<br>4. 1-2 days a week<br>5. 1-3 days a month<br>6. <1 day a month<br><br>-3. wrong skip<br>-2. not available<br>1. 11 glasses or more<br>2. 8-10 glasses<br>3. 6-7 glasses<br>4. 4-5 glasses<br>5. 2-3 glasses<br>6. 1 glass	If (sex=0 and emalcohd<=2 and emalcohn=6) → 2<br>If (sex=0 and emalcohd<=2 and emalcohn=5) → 3<br>If (sex=0 and emalcohd<=2 and emalcohn=4) → 4<br>If (sex=0 and emalcohd<=2 and (emalcohn=3 or emalcohn=2 or emalcohn=1)) → 2<br>If (sex=0 and emalcohd=3 and emalcohn=6) → 2<br>If (sex=0 and emalcohd=3 and emalcohn=5) → 2<br>If (sex=0 and emalcohd=3 and emalcohn=4) → 3<br>If (sex=0 and emalcohd=3 and emalcohn=3) → 4<br>If (sex=0 and emalcohd=3 and emalcohn=2) → 4<br>If (sex=0 and emalcohd=3 and emalcohn=1) → 5<br>If (sex=0 and emalcohd=4 and emalcohn=6) → 2<br>If (sex=0 and emalcohd=4 and emalcohn=5) → 2<br>If (sex=0 and emalcohd=4 and emalcohn=4) → 2<br>If (sex=0 and emalcohd=4 and emalcohn=3) → 3<br>If (sex=0 and emalcohd=4 and emalcohn=2) → 3<br>If (sex=0 and emalcohd=4 and emalcohn=1) → 3<br>If (sex=0 and emalcohd=5 and emalcohn=6) → 1<br>If (sex=0 and emalcohd=5 and (emalcohn=5 or emalcohn=4 or emalcohn=3 or emalcohn=2 or emalcohn=1)) → 2<br>If (sex=0 and emalcohd=6 and emalcohn>1) → 1<br>If (sex=0 and emalcohd=4 and emalcohn=1) → 2<br>If (sex=0 and emalcohu=1) → 0

			If (sex=1 and emalcohd<=2 and emalcohn=6) → 2<br>If (sex=1 and emalcohd=1 and emalcohn=5) → 4<br>If (sex=1 and emalcohd=1 and (emalcohn=4 or emalcohn=3 or emalcohn=2 or emalcohn=1)) → 5<br>If (sex=1 and emalcohd=2 and emalcohn=5) → 3<br>If (sex=1 and emalcohd=2 and (emalcohn=4 or emalcohn=3 or emalcohn=2 or emalcohn=1)) → 5<br>If (sex=1 and emalcohd=3 and emalcohn=6) → 2<br>If (sex=1 and emalcohd=3 and emalcohn=5) → 3<br>If (sex=1 and emalcohd=3 and emalcohn=4) → 4<br>If (sex=1 and emalcohd=3 and (emalcohn=3 or emalcohn=2 or emalcohn=1)) → 5<br>If (sex=1 and emalcohd=4 and emalcohn>=4) → 2<br>If (sex=1 and emalcohd=4 and emalcohn=3) → 3<br>If (sex=1 and emalcohd=4 and emalcohn=2) → 3<br>If (sex=1 and emalcohd=4 and emalcohn=1) → 4<br>If (sex=1 and emalcohd=5 and emalcohn=6) → 1<br>If (sex=1 and emalcohd=5 and (emalcohn=5 or emalcohn=4 or emalcohn=3 or emalcohn=2 or emalcohn=1)) → 2<br>If (sex=1 and emalcohd=6 and emalcohn>1) → 1<br>If (sex=1 and emalcohd=6 and emalcohn=1) → 2<br>If (sex=1 and emalcohu=1) → 0
Spain	Do you drink alcohol regularly (not just at parties or celebrations)? (alcohol_fr)<br><br><br>How often do you drink alcohol? (Alcohol)	1. yes<br>2. no<br>9. no answer<br><br>1. Less than 1/month<br>2. 1 or more times/month<br>3. at least 1 time/week<br>4. daily<br>5. it does not take anything<br>9. lost	All → -1

	How many days this past wek have you been drinking any alcoholic beverages? (drink_fr)	range: 0-7<br>99. missing	
UK	Alcohol (units per week) (halccat)	0. non-drinker<br>1. very low (0/<1 M&F)<br>2. low (1-10M, 1-7F)<br>3. moderate (11-21M, 8-14F)<br>4. fairly high (22-35M, 15-21 F)<br>5. high (>35M, >21 F)	copy
	**Smoking**	(smoke)	-1. missing<br>0. no<br>1. yes
Germany	Have you ever smoked at least 1 cigarette per day for one year (or a total of 20 packs in your life)? (IV1N146)<br><br>Did you quit smoking? (IV1N148)<br><br>How many cigarettes a day did you smoke, referring to the total time you smoked? (IV1N150)<br><br>Did you mainly smoke factory-made or hand-rolled cigarettes, referring to the total time you smoked? (IV1N151)<br><br>Have you ever smoked a pipe or cigars? (IV1N154)<br><br>Do you still smoke a pipe or cigars? (IV1N155)	1. yes<br>2. no<br><br>1. yes<br>2. no<br><br>range: 0-highest<br><br>1. factory-made<br>2. hand-rolled<br><br>1. yes<br>2. no<br><br>1. yes, pipe<br>2. yes, cigar<br>3. no	(IV1N146=1 and IV1N148=1) and (IV1NN154=1 and IV1N155=3) → 0<br>(IV1N146=1 and IV1N148=2) or (IV1NN154=1 and IV1N155<3) → 1<br>IV1N146=2 and IV1NN154=2 → 0
Italy	Cigarette smokers (SM1)<br>Pipe/cigars (SM7)	1. yes<br>2. no	SM1=1 or SM7=1 → 1<br>SM1=2 and SM7=2 → 0

	Number of cigarettes on average per day (SM2)	range: 0-highest	
Netherlands	Do you smoke? (emvar31)<br><br><br><br><br>Did you ever smoke before? (emvar32)<br><br><br><br><br>How many cigarettes or self-rolled cigarettes do you smoke a week on average? (emvar37)<br><br><br><br>How many pipes/cigars do you smoke a week on average? (emvar43)	-5. not available, interview terminated<br>-1. not available, asked<br>1. no<br>2. yes<br><br>-2. not available<br>1. no<br>2. yes<br><br>-2. not available<br>-1. not available, asked<br>range 0-highest<br><br>-2. not available<br>-1. not available, asked<br>range 0-highest	1 → 0<br>2 → 1<br>-5,-1 → -1
Spain	Summary variable smoker (smoker)	1. no smoke<br>2. ex-smoker for more than 7 years<br>3. ex-smoker for less than 7 years<br>4. occasionally (not daily)<br>5. fumador habitual (daily)<br>9. lost	1 → 0<br>2 → 0<br>3 → 0<br>4 → 1<br>5 → 5<br>9 → -1
UK	Smoker status (hsmkstat)<br><br><br><br>Current cigarettes per day (hccigs)<br>Current roll-up oz per week (hcrollup)<br>Current cigars per day (hccigar)<br>Current pipe tobacco oz per week (hctobac)	0. never<br>1. ex<br>2. current<br><br>range: 0-highest	0 → 0<br>1 → 0<br>2 → 1
	**Physical activity (continuous)**	(pa)	-1. missing<br>Range: 0-highest min/week
Germany	LAPAQ<br>Did you go for a walk during the past two weeks?	 Did you …. During the past two weeks?	Multiply frequency and duration per activity.<br>(Sum min/two weeks)/2

	(IV2N575)<br>- how many times (IV2N576)<br>- how long each time (IV2N577)<br><br>Did you cycle during the past two weeks? (IV2N580)<br>- how many times (IV2N581)<br>- how long each time (IV2N582)<br><br>Did you work in the garden during the past two weeks? (IV2N584)<br>- how many times (IV2N585)<br>- how long each time (IV2N586)<br><br>Do you do sports? (IV2N588)<br>During the past two weeks…<br>- which sport (IV2N589)<br>- how many times (IV2N591)<br>- how long each time (IV2N592)<br><br>Do you do a second sport? (IV2N593)<br>During the past two weeks…<br>- which sport (IV2N594)<br>- how many times (IV2N596)<br>- how long each time (IV2N597)<br><br>Do you do light household tasks? (IV2N599)<br>During the past two weeks…<br>- how many days (IV2N600)<br>- how long each day (IV2N601)<br><br>Do you do heavy household tasks? (IV2N602)<br>During the past two weeks…<br>- how many days (IV2N603)<br>- how long each day (IV2N604)	0. no<br>1. yes<br><br>Per activity, the time spend in minutes/day is calculated. The times spend on all activities are summed to express physical activity in minutes/day.	

Italy	Number of weekly hours devoted to:<br>- bowling (PA011)<br>- hunting/fishing (PA012)<br>- walking (PA013)<br>- dancing (PA014)<br>- cycling (PA015)<br>- gardening (PA016)<br>- gymnastics/fitness (PA017)<br>- swimming (PA018)	range: 0-highest	(Sum hours/week) *60.<br>Note that the time spend on household activities is not included!
Netherlands	Observation physical condition (elphya01)<br><br><br><br><br><br><br>Did you go for a walk during the past two weeks? (elphya07)<br>- how many times (elphya08)<br>- how long each time (elphya09)<br><br>Did you cycle during the past two weeks? (elphya11)<br>- how many times (elphya12)<br>- how long each time (elphya13)<br><br>Did you do sports during the past two weeks? (elphya21)<br>- which sport (elphya22)<br>- how many times (elphya23)<br>- how long each time (elphya24)<br><br>Did you do a second sport during the past two weeks? (elphya25)<br>- which sport (elphya26)	-5. not available, interview terminated<br>-4. not available, short interview<br>1. bedridden<br>2. in electric wheelchair<br>3. in mechanical wheelchair<br>4. not 1, 2 or 3<br><br>Did you …. During the past two weeks?<br>-3. not available, wrong skip<br>-2. not available<br>-1. not available, asked<br>1. no<br>2. yes<br><br>How many times: range 0-highest<br><br>How long each time (minutes): range 0-highest<br><br>Per activity, the time spend in minutes/day is calculated. The times spend on all activities are summed to express physical activity in minutes/day. Multiplying the times spend per activity with the MET-scores (Metabolic equivalent scores, adapted from Ainsworth, Compendium of physical activities: classification of energy costs of human physical activities,	Multiply frequency and duration per activity.<br>(Sum min/two weeks)/2

	- how many times (elphya27)<br>- how long each time (elphya28)<br><br>Did you do light household tasks during the past two weeks? (elphya32)<br>- how many days (elphya33)<br>- how long each day (elphya34)<br><br>Did you do heavy household tasks during the past two weeks? (elphya36)<br>- how many days (elphya37)<br>- how long each day (elph38)	Med.Sci.Sports Exerc. 1993; 25(1):71-80) and the respondents weight results in the kilocalories/day.	
Spain	PASE - hours per week:<br>- walking (PASE_walk)<br>- light sports (PASE_sport_light)<br>- moderate sports (PASE_sport_moder)<br>- vigorous sports (PASE_sport_vigor)<br>- light housework (PASE_house_light)<br>- heavy housework (PASE_house_heavy)<br>- care of garden (PASE_garden)<br>- caring for sick people (PASE_sick)<br>- caring for children (PASE_children)	Range: 0-highest (hours/week)	(Sum all activities)*60.
UK	PAQ: for a list of 69 activities (including walking, leisure /sports activities and housekeeping), it was reported if they did it (yes/no) and if yes, for what met. hr. energy expenditure per month (MET.h per month)	Did you ……<br>0. no<br>1. yes<br><br>MET.h per month: range: 0-highest	(((MET.h per month)/MET)/4)*60.
	**Physical activity (dichotomous)**	(pad)	-1. missing<br>0. no<br>1. yes
Germany	LAPAQ<br>Did you go for a walk during the past two weeks? (IV2N575)<br>- how many times (IV2N576)<br>- how long each time (IV2N577)	Did you …. During the past two weeks?<br>0. no<br>1. yes	Multiply frequency and duration per activity.<br>(Sum min/two weeks)/2<br>If min/week of physical activity>0 → 1.<br>If min/week of physical activity=0 → 0.

	Did you cycle during the past two weeks? (IV2N580)<br>- how many times (IV2N581)<br>- how long each time (IV2N582)<br><br>Did you work in the garden during the past two weeks? (IV2N584)<br>- how many times (IV2N585)<br>- how long each time (IV2N586)<br><br>Do you do sports? (IV2N588)<br>During the past two weeks…<br>- which sport (IV2N589)<br>- how many times (IV2N591)<br>- how long each time (IV2N592)<br><br>Do you do a second sport? (IV2N593)<br>During the past two weeks…<br>- which sport (IV2N594)<br>- how many times (IV2N596)<br>- how long each time (IV2N597)<br><br>Do you do light household tasks? (IV2N599)<br>During the past two weeks…<br>- how many days (IV2N600)<br>- how long each day (IV2N601)<br><br>Do you do heavy household tasks? (IV2N602)<br>During the past two weeks…<br>- how many days (IV2N603)<br>- how long each day (IV2N604)	Per activity, the time spend in minutes/day is calculated. The times spend on all activities are summed to express physical activity in minutes/day.	
Italy	Number of weekly hours devoted to:<br>- bowling (PA011)<br>- hunting/fishing (PA012)	range: 0-highest	(Sum hours/week) *60.<br>Note that the time spend on household activities is not included!

	- walking (PA013)<br>- dancing (PA014)<br>- cycling (PA015)<br>- gardening (PA016)<br>- gymnastics/fitness (PA017)<br>- swimming (PA018)		If min/week of physical activity>0 → 1.<br>If min/week of physical activity=0 → 0.
Netherlands	Observation physical condition (elphya01)<br><br><br><br><br><br><br>Did you go for a walk during the past two weeks? (elphya07)<br>- how many times (elphya08)<br>- how long each time (elphya09)<br><br>Did you cycle during the past two weeks? (elphya11)<br>- how many times (elphya12)<br>- how long each time (elphya13)<br><br>Did you do sports during the past two weeks? (elphya21)<br>- which sport (elphya22)<br>- how many times (elphya23)<br>- how long each time (elphya24)<br><br>Did you do a second sport during the past two weeks? (elphya25)<br>- which sport (elphya26)<br>- how many times (elphya27)<br>- how long each time (elphya28)	-5. not available, interview terminated<br>-4. not available, short interview<br>1. bedridden<br>2. in electric wheelchair<br>3. in mechanical wheelchair<br>4. not 1, 2 or 3<br><br>Did you …. During the past two weeks?<br>-3. not available, wrong skip<br>-2. not available<br>-1. not available, asked<br>1. no<br>2. yes<br><br>How many times: range 0-highest<br><br>How long each time (minutes): range 0-highest<br><br>Per activity, the time spend in minutes/day is calculated. The times spend on all activities are summed to express physical activity in minutes/day. Multiplying the times spend per activity with the MET-scores (Metabolic equivalent scores, adapted from Ainsworth, Compendium of physical activities: classification of energy costs of human physical activities, Med.Sci.Sports Exerc. 1993; 25(1):71-80) and the respondents weight results in the kilocalories/day.	Multiply frequency and duration per activity.<br>(Sum min/two weeks)/2<br><br>If min/week of physical activity>0 → 1.<br>If min/week of physical activity=0 → 0.

	Did you do light household tasks during the past two weeks? (elphya32)<br>- how many days (elphya33)<br>- how long each day (elphya34)<br><br>Did you do heavy household tasks during the past two weeks? (elphya36)<br>- how many days (elphya37)<br>- how long each day (elph38)		
Spain	PASE - hours per week:<br>- walking (PASE_walk)<br>- light sports (PASE_sport_light)<br>- moderate sports (PASE_sport_moder)<br>- vigorous sports (PASE_sport_vigor)<br>- light housework (PASE_house_light)<br>- heavy housework (PASE_house_heavy)<br>- care of garden (PASE_garden)<br>- caring for sick people (PASE_sick)<br>- caring for children (PASE_children)	Range: 0-highest (hours/week)	(Sum all activities)*60.<br><br>If min/week of physical activity>0 → 1.<br>If min/week of physical activity=0 → 0.
UK	PAQ: for a list of 69 activities (including walking, leisure /sports activities and housekeeping), it was reported if they did it (yes/no) and if yes, for what met. hr. energy expenditure per month (MET.h per month)	Did you ……<br>0. no<br>1. yes<br><br>MET.h per month: range: 0-highest	(((MET.h per month)/MET)/4)*60.<br><br>If min/week of physical activity>0 → 1.<br>If min/week of physical activity=0 → 0.
	**Physical activity (categorical)**	(pacat)	-1. missing<br>0. low<br>1. medium<br>2. high
Germany	LAPAQ<br>Did you go for a walk during the past two weeks? (IV2N575)<br>- how many times (IV2N576)<br>- how long each time (IV2N577)<br><br>Did you cycle during the past two weeks?	Did you …. During the past two weeks?<br>0. no<br>1. yes<br><br>Per activity, the time spend in minutes/day is calculated. The times spend on all activities are	(kcal/day)*7<br>Categorize into tertiles

	(IV2N580)<br>- how many times (IV2N581)<br>- how long each time (IV2N582)<br><br>Did you work in the garden during the past two weeks? (IV2N584)<br>- how many times (IV2N585)<br>- how long each time (IV2N586)<br><br>Do you do sports? (IV2N588)<br>During the past two weeks…<br>- which sport (IV2N589)<br>- how many times (IV2N591)<br>- how long each time (IV2N592)<br><br>Do you do a second sport? (IV2N593)<br>During the past two weeks…<br>- which sport (IV2N594)<br>- how many times (IV2N596)<br>- how long each time (IV2N597)<br><br>Do you do light household tasks? (IV2N599)<br>During the past two weeks…<br>- how many days (IV2N600)<br>- how long each day (IV2N601)<br><br>Do you do heavy household tasks? (IV2N602)<br>During the past two weeks…<br>- how many days (IV2N603)<br>- how long each day (IV2N604)	summed to express physical activity in minutes/day.	
Italy	Number of weekly hours devoted to:<br>- bowling (PA011)<br>- hunting/fishing (PA012)<br>- walking (PA013)<br>- dancing (PA014)	range: 0-highest	((Hours/week)*60)*MET<br>Categorize into tertiles

	- cycling (PA015)<br>- gardening (PA016)<br>- gymnastics/fitness (PA017)<br>- swimming (PA018)		
Netherlands	Observation physical condition (elphya01)<br><br><br><br><br><br><br>Did you go for a walk during the past two weeks? (elphya07)<br>- how many times (elphya08)<br>- how long each time (elphya09)<br><br>Did you cycle during the past two weeks? (elphya11)<br>- how many times (elphya12)<br>- how long each time (elphya13)<br><br>Did you do sports during the past two weeks? (elphya21)<br>- which sport (elphya22)<br>- how many times (elphya23)<br>- how long each time (elphya24)<br><br>Did you do a second sport during the past two weeks? (elphya25)<br>- which sport (elphya26)<br>- how many times (elphya27)<br>- how long each time (elphya28)<br><br>Did you do light household tasks during the past two weeks? (elphya32)	-5. not available, interview terminated<br>-4. not available, short interview<br>1. bedridden<br>2. in electric wheelchair<br>3. in mechanical wheelchair<br>4. not 1, 2 or 3<br><br>Did you …. During the past two weeks?<br>-3. not available, wrong skip<br>-2. not available<br>-1. not available, asked<br>1. no<br>2. yes<br><br>How many times: range 0-highest<br><br>How long each time (minutes): range 0-highest<br><br>Per activity, the time spend in minutes/day is calculated. The times spend on all activities are summed to express physical activity in minutes/day. Multiplying the times spend per activity with the MET-scores (Metabolic equivalent scores, adapted from Ainsworth, Compendium of physical activities: classification of energy costs of human physical activities, Med.Sci.Sports Exerc. 1993; 25(1):71-80) and the respondents weight results in the kilocalories/day.	(kcal/day)*7<br>Categorize into tertiles

	- how many days (elphya33)<br>- how long each day (elphya34)<br><br>Did you do heavy household tasks during the past two weeks? (elphya36)<br>- how many days (elphya37)<br>- how long each day (elph38)		
Spain	PASE - hours per week:<br>- walking (PASE_walk)<br>- light sports (PASE_sport_light)<br>- moderate sports (PASE_sport_moder)<br>- vigorous sports (PASE_sport_vigor)<br>- light housework (PASE_house_light)<br>- heavy housework (PASE_house_heavy)<br>- care of garden (PASE_garden)<br>- caring for sick people (PASE_sick)<br>- caring for children (PASE_children)	Range: 0-highest (energy expenditure/week)	COMPUTE PASE_proxy =<br>PASE_walk +<br>PASE_sport_light +<br>(2 * PASE_sport_moder) +<br>(3 * PASE_sport_vigor) +<br>PASE_house_light +<br>(2 * PASE_house_heavy) +<br>(2 * PASE_garden) +<br>(2 * PASE_sick) +<br>(2 * PASE_children) .<br><br>Categorize into tertiles
UK	PAQ: for a list of 69 activities (including walking, leisure /sports activities and housekeeping), it was reported if they did it (yes/no) and if yes, for what met. hr. energy expenditure per month (MET.h per month)	Did you ……<br>0. no<br>1. yes<br><br>MET.h per month: range: 0-highest	(MET.h per month)/30<br>Categorize into tertiles
	**Walking**	(walking)	-1. missing<br>Range: 0-highest Min/week
Germany	LAPAQ<br>Did you go for a walk during the past two weeks? (IV2N575)<br>- how many times (IV2N576)<br>- how long each time (IV2N577)	0. no<br>1. yes	Min/week
Italy	Number of weekly hours devoted to:<br>- walking (PA013)	range: 0-highest	Min/week
Netherlands	Observation physical condition (elphya01)	-5. not available, interview terminated<br>-4. not available, short interview	Min/week

	Did you go for a walk during the past two weeks? (elphya07)<br>- how many times (elphya08)<br>- how long each time (elphya09)	1. bedridden<br>2. in electric wheelchair<br>3. in mechanical wheelchair<br>4. not 1, 2 or 3<br><br>Did you …. During the past two weeks?<br>-3. not available, wrong skip<br>-2. not available<br>-1. not available, asked<br>1. no<br>2. yes<br><br>How many times: range 0-highest<br>How long each time (minutes): range 0-highest	
Spain	PASE:<br>Frequency and time of:<br>- walking (PASE_walk_fr, PASE_walk_t)	0. never<br>1. <1 week<br>2. 1-2/week<br>3. 3-4/week<br>4. 5-7/week<br>9. no answer<br><br>1. <30 min every time<br>2. 30-60 min every time<br>3. >60 min every time<br>9. no answer	Take the "average" frequency and time per category. ">60 min" is interpreted as 60 or more.<br><br>If walk_fr=0 → 0<br>If walk_fr=1 and walk_t=1 → 0.5*15<br>If walk_fr=1 and walk_t=2 → 0.5*45<br>If walk_fr=1 and walk_t=3 → 0.5*60<br>If walk_fr=2 and walk_t=1 → 1.5*15<br>If walk_fr=2 and walk_t=2 → 1.5*45<br>If walk_fr=2 and walk_t=3 → 1.5*60<br>If walk_fr=3 and walk_t=1 → 3.5*15<br>If walk_fr=3 and walk_t=2 → 3.5*45<br>If walk_fr=3 and walk_t=3 → 3.5*60<br>If walk_fr=4 and walk_t=1 → 6*15<br>If walk_fr=4 and walk_t=2 → 6*45<br>If walk_fr=4 and walk_t=3 → 6*60<br>If walk_fr=9 or walk_t=9 → -1
UK	Walking outdoors (minutes per day):<br>- continuous (hwlktot)	Did you ……<br>0. no<br>1. yes	Min/week

		range: 0-600	
	**Sports activities**	(sports)	-1. missing<br>0. no<br>1. yes
Germany	Do you do sports? (IV2N588)<br>During the past two weeks…<br>- which sport (IV2N589)<br>- how many times (IV2N591)<br>- how long each time (IV2N592)<br><br>Do you do a second sport? (IV2N593)<br>During the past two weeks…<br>- which sport (IV2N594)<br>- how many times (IV2N596)<br>- how long each time (IV2N597)	0. no<br>1. yes<br><br>Per activity, the time spend in minutes/day is calculated. The times spend on all activities are summed to express physical activity in minutes/day.	If yes on IV2N588 or IV2N593 → 1<br>If no on IV2N588 and IV2N593 → 0
Italy	Number of weekly hours devoted to:<br>- bowling (PA011)<br>- hunting/fishing (PA012)<br>- dancing (PA014)<br>- cycling (PA015)<br>- gymnastics/fitness (PA017)<br>- swimming (PA018)	range: 0-highest	If PA011>0 or PA12>0 or PA14>0 or PA15>0 or PA17>0 or PA18>0 → 1<br>If all 0 → 0
Netherlands	Did you do sports during the past two weeks? (elphya21)<br>- which sport (elphya22)<br>- how many times (elphya23)<br>- how long each time (elphya24)<br><br>Did you do a second sport during the past two weeks? (elphya25)<br>- which sport (elphya26)<br>- how many times (elphya27)<br>- how long each time (elphya28)	Did you …. During the past two weeks?<br>-3. not available, wrong skip<br>-2. not available<br>-1. not available, asked<br>1. no<br>2. yes	If yes on elphya21 or elphya25 → 1<br>If no on elphya21 and elphya25 → 0<br>If missing (elphya21) → -1
Spain	PASE:	0. never	If PASE_sport_light_fr>0 or

	Frequency and time of:<br>- light sports (PASE_sport_light_fr, PASE_sport_light_t)<br>- moderate sports (PASE_sport_moder_fr, PASE_sport_moder_t)<br>- vigorous sports (PASE_sport_vigor_fr, PASE_sport_vigor_t)	1. <1 week<br>2. 1-2/week<br>3. 3-4/week<br>4. 5-7/week<br>9. no answer	PASE_sport_moder_fr>0 or PASE_sport_vigor_fr>0 → 1<br>If PASE_sport_light_fr=0 and PASE_sport_moder_fr=0 and PASE_sport_vigor_fr=0 → 0<br>If PASE_sport_light_fr=9 and PASE_sport_moder_fr=9 and PASE_sport_vigor_fr=9 → -1
UK	PAQ: for a list of 69 activities (including walking, leisure /sports activities and housekeeping), it was reported if they did it (yes/no) and if yes, for what met. hr. energy expenditure per month (MET.h per month)	Did you ……<br>0. no<br>1. yes	If yes on any sports/exercise activity → 1<br>If no on all sports/exercise activity → 0.<br>If missing on all sports/exercise activity →-1
	**Diet/nutrition**		Too heterogeneous, not possible to harmonize
Germany	Mini Nutritional Assessment (MNA)<br>(no total score variable generated yet)	range: 0-30<br>Can be classified into 3 categories: normal, at risk and bad condition	
Italy	Reason weight change last year:<br>- diet (WE041)<br>- physical movement (WE042)<br>- surgery/illness (WE043)<br>- other (WE044)	1. no<br>2. yes<br>9. don’t know	
Netherlands	Not available		
Spain	Not available		
UK	All dietary food items are in terms of "weekly equivalent frequencies" except for milk (pints/day) and sugar (teaspoons/day). Corrections for portion size have been applied to variables as necessary. The 126 food items were combined into 54 food groups (dfgrp1-dfgrp54)	range: 0-highest	

PHYSICAL FUNCTIONING

STUDY	QUESTION (variable name)	VALUE	EPOSA CODE
	**5 times chair stands**	(chair)	-1. missing<br>Range 0-highest (seconds)
Germany	Time in seconds needed to perform five rises from a chair to an upright position as fast as possible without the use of the arms (IV1N367)	range: 0-highest<br>99. Not applicable	Range 0-highest
Italy	Chair test: seconds needed to rise 5 times (CTB2)	range: 0-highest	Range 0-highest
Netherlands	Time in seconds needed to stand up from a kitchen chair without use of arms (echair7)	-6. not available, no measurement<br>-2. not available, unable<br>-1. no valid answer<br>range 0-highest	Range 0-highest
Spain	chair rises:<br>- able to (BAL5_A)<br>- time (secs) (BAL5_C)	1. yes could do<br>2. no<br>3. not applicable<br><br>Range: 0-highest	Range 0-highest
UK	chair rises: 5 rises done (crfive)<br><br><br>chair rises: time (secs) (crtime)	0. no<br>1. yes<br><br>range: 0-highest	Range 0-highest
	**5 times chair stands, categorical**	(chairc)	-1. missing<br>0. unable<br>1. poor<br>2. fair<br>3. good<br>4. excellent
Germany	Time in seconds needed to perform five rises from a chair to an upright position as fast as possible without the use of the arms (IV1N367)	range: 0-highest<br>99. Not applicable	Create quartiles of chair. Fastest quartile indicates excellent performance<br>If (chair=-1) → 0<br>Q1 → 4<br>Q2 → 3

			Q3 → 2<br>Q4 → 1
Italy	Chair test: seconds needed to rise 5 times (CTB2)	range: 0-highest	Create quartiles of chair. Fastest quartile indicates excellent performance<br>If (chair=-1) → 0<br>Q1 → 4<br>Q2 → 3<br>Q3 → 2<br>Q4 → 1
Netherlands	Time in seconds needed to stand up from a kitchen chair without use of arms (echair7)	-6. not available, no measurement<br>-2. not available, unable<br>-1. no valid answer<br>range 0-highest	Create quartiles of chair. Fastest quartile indicates excellent performance<br>If (chair=-1) → 0<br>Q1 → 4<br>Q2 → 3<br>Q3 → 2<br>Q4 → 1
Spain	chair rises:<br>- able to (BAL5_A)<br>- time (secs) (BAL5_C)	1. yes could do<br>2. no<br>3. not applicable<br><br>Range: 0-highest	Create quartiles of chair. Fastest quartile indicates excellent performance<br>If (chair=-1) → 0<br>Q1 → 4<br>Q2 → 3<br>Q3 → 2<br>Q4 → 1
UK	chair rises: 5 rises done (crfive)<br><br><br>chair rises: time (secs) (crtime)	0. no<br>1. yes<br><br>range: 0-highest	Create quartiles of chair. Fastest quartile indicates excellent performance<br>If (chair=-1) → 0<br>Q1 → 4<br>Q2 → 3<br>Q3 → 2<br>Q4 → 1
	**Walk test**	(walk)	-1. missing<br>Range: 1-10
Germany	Length of distance to walk? (IV1N358)<br>(4 meters if possible given the space in the home, if not then 3 meters)	1. four meters<br>2. three meters	If 4m, categorize first attempt into deciles<br>If 3 m, categorize first attempt into deciles

	Time in seconds needed to walk three/four meters at a normal pace (2 measurements) (IV1N360, IV1N361).	range: 0-highest	
Italy	Timed walk:<br>Length of distance to walk (TMA1)<br><br>Time needed to walk three/four meters (TMA31)	1. 4 m.<br>2. 3 m.<br>3. not applicable<br>range: 0-highest	If 4m, categorize first attempt into deciles<br>If 3 m, categorize first attempt into deciles
Netherlands	Time in seconds needed to walk 3 meters along a line, turn 360° and walk back (ewalk04)	-6. not available, no measurement<br>-2. not available, unable<br>-1. no valid answer<br>range 0-highest	Categorize ewalk04 into deciles
Spain	Time needed to walk three/four meters<br>- able to (BAL4_A)<br>- distance (BAL4_B)<br>- time in seconds (BAL4_C)	1. yes could do<br>2. no<br>3. not applicable<br><br>1. four meters<br>2. three meters<br>3. not applicable<br><br>Range: 0-highest	If 4m, categorize into deciles<br>If 3 m, categorize into deciles
UK	6 m timed up and go (sec) (tu6mupgo)<br><br>3m walk (sec) (tu3mwlk)	range: 0-highest<br><br>range: 0-highest	Categorize tu3mwlk into deciles
	**Walk test, categorical**	(walkc)	-1. missing<br>0. unable<br>1. poor<br>2. fair<br>3. good<br>4. excellent
Germany	Length of distance to walk? (IV1N358)<br>(4 meters if possible given the space in the home, if not then 3 meters)<br><br>Time in seconds needed to walk three/four meters at	1. four meters<br>2. three meters<br><br><br>range: 0-highest	Recode walk into 4 categories:<br>-1 →-1<br>Categories depend on data

	a normal pace (2 measurements) (IV1N360, IV1N361).		
Italy	Timed walk:<br>Length of distance to walk (TMA1)<br><br>Time needed to walk three/four meters (TMA31)	1. 4 m.<br>2. 3 m.<br>3. not applicable<br>range: 0-highest	-1 →-1<br>0, 1, 2, → 4<br>3, 4 → 3<br>5, 6, 7 → 2<br>≥8 → 1
Netherlands	Time in seconds needed to walk 3 meters along a line, turn 360° and walk back (ewalk04)	-6. not available, no measurement<br>-2. not available, unable<br>-1. no valid answer<br>range 0-highest	-1 →-1<br>0, 1, 2, → 4<br>3 → 3<br>4,5 → 2<br>≥6 → 1
Spain	Time needed to walk three/four meters<br>- able to (BAL4_A)<br>- distance (BAL4_B)<br>- time in seconds (BAL4_C)	1. yes could do<br>2. no<br>3. not applicable<br><br>1. four meters<br>2. three meters<br>3. not applicable<br><br>Range: 0-highest	-1 →-1<br>0, 1, 2 → 4<br>3,4 →3<br>5, 6, 7 →2<br>≥8 → 1
UK	6 m timed up and go (sec) (tu6mupgo)<br><br>3m walk (sec) (tu3mwlk)	range: 0-highest<br><br>range: 0-highest	-1 →-1<br>0, 1, 2→ 4<br>3,4,5 →3<br>6, 7 →2<br>≥8 → 1
	**Tandem stand**	(tandem)	-1. missing<br>0. unable<br>1. poor performance<br>2. good performance
Germany	Being able to balance for more than ten seconds:	0. no	If (IV1N352=1) → 2

	- side-by-side stand (IV1N350)<br>- semi-tandem stand (IV1N351)<br>-tandem stand (IV1N352)<br><br>If "no" on IV1N352, for how many seconds? (IV1N353)	1. yes<br>2. not conducted<br><br><br>range: 0-10	If (IV1N352=0 and IV1N353<4) → 0<br>If (IV1N353>3 and IV1N353<10) → 1<br>IV1N352=2 → -1<br>If (IV1N352=0 and sysmis(IV1N352) → -1
Italy	Balance test: seconds (BTA11)	range: 0-10	Sysmis(BTA11) → -1<br>BTA11<4 → 0<br>BTA11>3 and BTA11<10 → 1<br>BTA11=10→ 2
Netherlands	Tandem stand: able to<br><br>Time in seconds able to hold the tandem stand, i.e. one foot in front of the other, heel against toe (etandem2)	-6. not available, test not done<br>-3. not available, wrong skip<br>-2. not available<br>-1. not available, asked<br>1. normal test<br>2. not capable<br>3. falls almost directly<br>4. stops within 3 sec.<br>5. refusal<br>6. physically impossible<br><br>-6. not available, no measurement<br>-2. not available, unable<br>-1. no valid answer<br>Range: 4-10	etandem1<0 → -1<br>If (etandem1>1) → 0<br>If (etandem1=5) → -1<br>etandem2>3 and etandem2<10 → 1<br>etandem2=10 → 2
Spain	Tandem stand<br>- able to (BAL3_A)<br>- time in seconds (BAL3_B)	1. yes could do<br>2. no<br>3. not applicable<br>99. missing<br><br>Range: 0-highest<br>99. missing	If (BAL3_A=2) → 0<br>If (BAL3_A=1 and BAL3_B<4) → 0<br>If (BAL3_A=1 and BAL3_B>3 and BAL3_B<10) → 1<br>If (BAL3_A=1 and BAL3_B>=10) → 2<br>If (BAL3_A=3) → -1<br>If (BAL3_A=1 and BAL3_B=99) → 2
UK	Flamingo stands: time in seconds to hold a one-leg stance for a maximum of 30 sec (fstime)	range: 0.00-30.00	If sysmis(fstime) → -1<br>fstime<=3 → 0<br>fstime>3 and fstime<10 → 1

			fstime>=10 → 2
	**Physical performance score**	(tpps)	-1. missing<br>Range: 0-10.
Germany<br>Italy<br>Netherlands<br>Spain<br>UK			The scores of the chair stands and walk test range from 1 (slowest) to 4 (fastest), corresponding to the quartiles of time needed in that cohort. The score of 0 was assigned when unable to complete the test. The tandem stand was categorized as described above (0,1,2 points). The total physical performance score is the sum of the three tests.
	**Functional limitations/ADL**	A combination of the variables functional limitations, ADL and IADL was made because not all variables were available in all countries and the content of the variables was largely overlapping (adl).<br>Note that the activities included differ across countries; however, we tried to match the activities as much as possible according to their underlying physical demands.	Per item (stairs, toenails, dress, rising, shower, fingers, highshelf, shop)<br>-1. missing<br>0. no difficulty<br>1. with difficulty<br>2. unable to do alone<br><br>Sum score: range: 0-10
Germany	What difficulties do you have when you want to:<br>-walk up and down a staircase of 15 steps without having to stop? (IV1N371)<br>-cut your toe nails? (IV1N372)<br>-dress or undress yourself? (IV1N373)<br>-sit down on and rise from a chair? (IV1N374)<br>-walk outside for five minutes without having to stand still? (IV1N375)<br>-use your own or public transportation? (IV1N377)<br>-take a shower or bath? (IV1N376)<br>-go shopping yourself? (IV1N378)<br>-prepare and take your medication? (IV1N379)<br>-do light housework yourself? (IV1N380)	1. none<br>2. some difficulties<br>3. moderate difficulties<br>4. much difficulties<br>5. I cannot, I need help	-walk up and down a staircase of 15 steps without having to stop? (IV1N371)<br>-cut your toe nails? (IV1N372)<br>-dress or undress yourself? (IV1N373)<br>-sit down on and rise from a chair? (IV1N374)<br>-take a shower or bath? (IV1N376)<br><br>1 → 0<br>2 ,3,4 → 1<br>5 → 2

Italy	Difficulty to (without help from a person or support):<br>-move in and out of bed or chair (ADL011, ADL012)<br>-walking into a room (ADL021, ADL022)<br>-climbing a flight of stairs (ADL031, ADL032)<br>-bathing or showering (ADL041, ADL042)<br>-dressing or undressing (ADL051, ADL052)<br>-eating and using cutlery (ADL061, ADL062)<br>-using the toilet (ADL071, ADL072)<br>-raising arms above the head (ADL081, ADL082)<br>-using fingers to catch and handle small things (ADL091, ADL092)<br>-lifting and carrying a weight of >5kg (ADL101, ADL102)	Difficulty:<br>1. yes<br>2. no<br><br>Degree of difficulty:<br>1. little difficult<br>2. difficult<br>3. very difficult<br>4. impossible	-move in and out of bed or chair (ADL011, ADL012)<br>-climbing a flight of stairs (ADL031, ADL032)<br>-bathing or showering (ADL041, ADL042)<br>-using fingers to catch and handle small things (ADL091, ADL092)<br>-raising arms above the head (ADL081, ADL082)<br><br>If difficulty=2 → 0<br>If difficulty=1 and degree=1,2,3 → 1<br>If difficulty=1 and degree=4 →2
Netherlands	Sum of scores on 6 items:<br>- Are you able to walk up and down a staircase of 15 steps without having to stop? (eadl1a)<br>- Are you able to dress and undress yourself? (eadl2a)<br>- Are you able to sit down and rise from a chair? (eadl3a)<br>- Are you able to cut your own toenails? (eadl4a)<br>- Are you able to walk outside for five minutes without having to stand still? (eadl5a)<br>- Are you able to use your own or public transportation? (eadl6a)<br><br>EQ-5D: self care (eqeq5d2)	-5. not available, interview terminated<br>-2. not available<br>-1. not available, asked<br>1. not available, I cannot<br>2. only with help<br>3. yes, with much difficulty<br>4. yes, with some difficulty<br>5. yes, without help<br><br>1. no problems<br>2. some problems<br>3. unable to	- Are you able to walk up and down a staircase of 15 steps without having to stop? (eadl1a)<br>- Are you able to dress and undress yourself? (eadl2a)<br>- Are you able to sit down and rise from a chair? (eadl3a)<br>- Are you able to cut your own toenails? (eadl4a)<br>- EQ-5D: self care (eqeq5d2)<br><br><0 → -1<br>1,2 → 2<br>3,4 → 1<br>5 → 0<br><br>EQ-5D: self care<br>1 → 0<br>2 → 1<br>3 → 2
Spain	Sum of scores on 7 items:	Per item:	- How difficult is it for you to pick up or

	- How difficult is it for you to pull or push a large object, such as an armchair? (FL_armchair)<br>- How difficult is it for you to bend over, to crouch or to kneel down? (FL_Kneel)<br>- How difficult is it for you to stretch the arms up above your shoulders? (FL_arms)<br>- How difficult is it for you to pick up or handle small objects? (FL_pick_up)<br>- How difficult is it for you to carry bags of less than 5 kg? (FL_bags)<br>- How difficult is it for you to go up and down stairs? (FL_stairs)<br>- How difficult is it for you to walk 1 km? (FL_walk)<br><br>- Are you able to walk through a small room? (ADL_Walk)<br>- Are you able to take a shower? (ADL_Shower)<br>- Are you able to tidy yourself up? (ADL_Tidy)<br>- Are you able to get dressed? (ADL_Dress)<br>- Are you able to eat alone? (ADL_Eat)<br>- Are you able to get out of bed? (ADL_Bed)<br>- Are you able to stand up from the seat? (ADL_Seat)<br>- Are you able to use the toilet? (ADL_Toilet)	1. no difficulty<br>2. any difficulty<br>3. more difficulty<br>4. unable<br>9. missing<br><br>Sum score range: 0-7.<br>Difficulty (yes/no): 1 point per item ≥2<br>OR<br>Able (yes/no): 1 point per item =4	handle small objects? (FL_pick_up)<br>- How difficult is it for you to go up and down stairs? (FL_stairs)<br>- Are you able to take a shower? (ADL_Shower)<br>- Are you able to stand up from the seat? (ADL_Seat)<br>- How difficult is it for you to stretch the arms up above your shoulders? (FL_arms)<br><br>1 → 0<br>2,3 → 1<br>4 → 2<br>9 → -1
UK	Townsend Disability scale (shortened):<br>Difficulty:<br>- washing down (fqwash)<br>- removing jug from high shelf (fqjug)<br>- tying a good knot in string (fqknot)<br>- cutting toenails (fqtoe)<br>- running to catch a bus (fqrunbus)<br>- going up/down stairs (fqstair)<br>- shopping and carrying basket (fqshop)<br>- doing heavy household work (fqhouswk)<br>- preparing a hot meal (fqmeal)	0. none<br>1. some difficulty<br>2. unable to do alone	- washing down (fqwash)<br>- removing jug from high shelf (fqjug)<br>- cutting toenails (fqtoe)<br>- going up/down stairs (fqstair)<br>- tying a good knot in string (fqknot)<br><br>0 → 0<br>1 → 1<br>2 → 2
	**(I)ADL**		Available in Italy and Spain

Germany	See functional limitations		
Italy	Difficulty:<br>-writing (IADL011, IADL012)<br>-preparing food (IADL021, IADL022)<br>-doing light work at home (IADL031, IADL032)<br>-difficulty to use telephone (IADL041, IADL042)<br>-difficulty keeping the household accounts (IADL051, IADL052)<br>-difficulty in driving a car (IADL061, IADL062)<br>-difficulty to travel on public transportation (IADL071, IADL072)<br>-difficulty opening a lock with a key (IADL081, IADL082)<br>-difficulty for taking medication at correct time (IADL091, IADL092)<br>-difficulty to kneel or bend down (IADL101, IADL102)	Difficulty:<br>1. yes<br>2. no<br><br>Degree of difficulty:<br>1. little difficult<br>2. difficult<br>3. very difficult<br>4. impossible	
Netherlands	See functional limitations		
Spain	Sum scores on 8 items:<br>- Are you able to use the telephone? (IADL_Phone)<br>- Are you able to catch the bus? (IADL_bus)<br>- Are you able to buy food? (IADL_buy)<br>- Are you able to go out on an errand? (IADL_errand)<br>- Are you able to cook? (IADL_cook)<br>- Are you able to do the light work of the house? (IADL_lightw)<br>- Are you able to do the heavy work of the house? (IADL_heavyw)<br>- Are you able to manage your own money? (IADL_money)<br>- Are you able to drive or take drugs prescribed for you? (IADL_drugs)	Per item:<br>1. able<br>2. help in any<br>3. unable<br>9. missing<br><br>Sum score range: 0-8.<br>Independence (yes/no): 1 point per item =1	
UK	See functional limitations		
	**Days of limited activity**	Not available in 3 or more cohorts	Available in NL

	**Gait pattern**	Not available in 3 or more cohorts	Available in Germany and NL
Germany	Questions to interviewer: How would you rate the gait balance of the participant? (IV1N362)	1. very good (walking straight and stable)<br>2. good<br>3. moderate<br>4. bad (unsteady and fluctuating)	
Italy	-use a stick or crutches to help him walk (ADL121)<br>-using a wheelchair to move (ADL131)<br>-walks leaning on a person (ADL141)<br>-walking leaning on the furniture of the house (ADL151)	1. yes<br>2. no<br>3. not walk<br>9. don't know	
Netherlands	Interviewer's observations during walk test:<br>- no particularities (ewalk09)<br>- problems starting (ewalk10)<br>- unstable gait (ewalk11)<br>- limps (ewalk12)<br>- has stiff legs (ewalk13)<br>- slides (ewalk14)<br>- trains with one leg (ewalk15)<br>- unstable turn (ewalk16)<br>- walks irregular (ewalk17)<br>- other (ewalk18)	-2. not available<br>0. not mentioned<br>1. mentioned	
Spain	Not available		
UK	Degree of walking problems (hwlkprob)	0. no limiting abnormality<br>1. abnormal gait/walking problems, no aid<br>2. using walking aid<br>3. requires help form another person<br>4. unable to walk	
	**Walking aid**	(walkaid)	-1. missing<br>0. no<br>1. yes
Germany	Walking aid during walk test (IV1N359)	1. none<br>2. one walking stick<br>3. two walking sticks/crutches<br>4. walking frame	1 → 0<br>2,3,4 → 1

Italy	Use a stick or crutches to help him walk (ADL121)	1. yes<br>2. no<br>3. not walk<br>9. don't know	1 → 1<br>2 → 0<br>3,9 → -1
Netherlands	Walking aid during walk test (ewalk06)	-2. not available<br>1. walkingbar<br>2. stick<br>3. rollator<br>4. leaning on objects/interviewer<br>5. other<br>6. none	-2 → -1<br>1,2,3 → 1<br>4 → 0
Spain	Not available		
UK	Walking aid during time up and go (tuwlkaid)	0. none<br>1. stick<br>2. frame<br>3. other	0 → 0<br>1,2,3 → 1
	**Grip strength**	(gripl, gripr)	Range: 0-highest (kg)
Germany	Hand grip strength is measured in kilograms using the JAMAR dynamometer. Both hands measured twice (IV3N803, IV3N804, IV3N805, IV3N806)	range: 1-highest	Mean of 2 trials per hand
Italy	test with right hand: first (kg) (GS51)<br>test with right hand: second (kg) (GS52)<br>test with right hand: third (kg) (GS53)<br>test with left hand: first (kg) (GS61)<br>test with left hand: second (kg) (GS62)<br>test with left hand: third (kg) (GS63)	range: 1-highest	Mean of first 2 trials per hand
Netherlands	Hand grip strength was measured in kilograms using a strain-gauged dynamometer (Takei TKK 5001, Takei Scientific Instruments Co. Ltd., Tokyo, Japan): both hand were measured twice. (emed616, emed617, emed618 emed619)	-2. not available<br>-1. no valid data<br>range: 0-highest	Mean of 2 trials per hand
Spain	Not available		All missing
UK	Hand grip strength was measured in kilograms using the JAMAR dynamometer. Both hands were	range: 0-highest	Mean of first 2 trials per hand

	measured 3 times. (agripr1 agripr2 agripr3 agripl1 agripl2 agripl3)		
	**Knee extensor strength**	Not available in 3 or more cohorts	Available in Italy

SUBJECTIVE HEALTH

STUDY	QUESTION (variable name)	VALUE	EPOSA CODE
	**Quality of life**	(EQ, PCS MCS)	The SF-12 and SF-36 will be pooled, the Euroqol and SF12/36 will not be harmonized.
Germany	SF-12 (no total score generated yet)		Copy
Italy	Not available		
Netherlands	Sf-12<br>- physical component<br>- mental component<br><br>EuroQol (lasae335)	-1. no valid data<br>range: 0-100<br><br><br>-2. no valid score<br>range: 0-1	SF-12: Copy<br><br><br><br>Euroquol: with uk-weights
Spain	Not available		
UK	Euroquol<br>- mobility (kqeumob)<br>- self care (kqeucare)<br>- usual activities (kqeuact)<br><br>- pain/discomfort (kqeupain)<br>- anxiety/depression (kqeuanx)<br><br>SF36:<br>- physical health summary (hsf36p)<br>- mental health summary (hsf36m)	 1. no problem<br>2. some problem<br>3. confined/unable to perform<br><br>1. no pain/anxious<br>2. moderate pain/anxious<br>3. extreme pain/anxious<br><br>range: 0-100	Euroquol: with uk-weights<br><br>SF-36: Select the 12 questions from the SF-12 and compute the component scores.
	**Pain**	(pain)	-1. missing<br>1. not at all<br>2. mild<br>3. moderate<br>4. severe<br>5. very severe
Germany	Structured Pain Interview (Basler et al.,		

	2001), consisting of<br>a) Locations of pain: (IV3N736 – IV3N749)<br>- mouth/teeth<br>- head/face<br>- neck<br>- shoulder/arm/hand<br>- chest<br>- upper spine<br>- belly<br>- lower spine<br>- hip/leg/foot<br>- pelvis<br>- sexual organs<br>- multiple joints<br>- whole body<br>- other areas<br><br>b) Pain intensity (IV3N751)<br>How intense was your pain where it hurt the most during the past seven days?<br><br>c) Determination of chronic pain:<br>For how long have you been feeling the pain that hurts the most? (IV3N753)<br><br>What character is your pain? Constant or on and off? (IV3N754)<br><br>During the past seven days, how often have you been troubled by pain? (IV3N755)	0. no<br>1. yes<br><br>range: 0 (no pain) – 10 (severe pain)<br>99. no answer<br><br>1. for days<br>2. for weeks<br>3. for months<br>4. for years<br><br>1. on and off, no pain in between<br>2. constant pain<br>3. constant pain, additional pain attacks<br><br>0. never<br>1. rarely<br>2. sometimes<br>3. frequently<br>4. very often	

	SF-12: Have you been limited in your daily activities by pain during the past 4 weeks (..)	5. always<br><br>1. not at all<br>2. mild<br>3. moderate<br>4. severe<br>5. very severe	copy
Italy	Pain or difficulty moving (in the last year):<br>- lumbar spine (AR15)<br>- hand or wrists (AR18)<br>- shoulders (AR21)<br>- hips (AR24)<br>- knees (AR28)<br>- ankles or feet (AR31)<br><br>Pain or difficulty moving (last month):<br>- lumbar spine (AR16)<br>- hand or wrists (AR19)<br>- shoulders (AR22)<br>- hips (AR25)<br>- knees (AR29)<br>- ankles or feet (AR32)<br><br>Type of pain/difficulty of movement	1. yes<br>2. no<br>9. don't know<br><br>Dito<br><br>1. yes, less than 1 week<br>2. yes, 1 or 3 weeks<br>3. yes, almost 1 month<br>4. yes, more than 1 month<br>5. no<br>9. don't know	missing
Netherlands	Subjective pain scale (Nottingham health profile, Hunt et al, 1985) (eqpain)<br><br>SF-12: Have you been limited in your daily activities by pain during the past 4 weeks (..)	-1. no valid data<br>range: 5 (no pain) – 10 (severe pain)<br><br>1. not at all<br>2. mild<br>3. moderate	 <br>copy

		4. severe<br>5. very severe	
Spain	Not available		
UK	SF36: bodily pain during past 4 weeks (hbodily)<br><br><br><br><br><br><br>WOMAC pain score (knee) (kqwomac)<br><br>Hand: any pain right and left hand (herp, help)	1. very severe<br>2. severe<br>3. moderate<br>4. mild<br>5. very mild<br>6. none<br><br>range: 0-18<br><br>0. no<br>1. yes	1 → 5<br>2 → 4<br>3 → 3<br>4,5 → 2<br>6 → 1
	**Self perceived health**	(sph)	-1. missing<br>1. bad<br>2. fair<br>3. good/excellent
Germany	Self-perceived health (IV1N394)	1. excellent<br>2. very good<br>3. good<br>4. suboptimal<br>5. poor	1,2,3 → 3<br>4 → 2<br>5 → 1
Italy	Self-perceived health (SPH1)	1. good/excellent<br>2. so-so<br>3. bad	1 → 3<br>2 → 2<br>3 → 1
Netherlands	Self-perceived health (esubhea1)	-5. not available<br>1. excellent<br>2. good<br>3. fair<br>4. sometimes good/bad<br>5. poor	-5 → -1<br>1,2 → 3<br>3,4 → 2<br>5 → 1
Spain	How would you describe your health today? (Health_today)	1. very good<br>2. good	1,2 → 3<br>3 → 2

		3. fair<br>4. poor<br>5. very poor<br>9. missing	4,5 → 1<br>9 → -1
UK	SF36: General health status (hgenhlth)	1. poor<br>2. fair<br>3. good<br>4. very good<br>5. excellent	1 → 1<br>2 → 2<br>3,4,5 → 3

SOCIAL FUNCTIONING

	**Social participation**		Too heterogeneous, not possible to harmonize
Germany	6-item Lubben Social Network Scale<br>(no total score variable generated yet)<br><br>Considering the people to whom you are related either by birth or marriage…<br>1. How many relatives do you see or hear from at least once a month?<br>2. How many relatives do you feel at ease with that you can talk about private matters?<br>3. How many relatives do you feel close to such that you could call on them for help?<br><br>Considering all of your friends including those who live in your neighborhood….<br>4. How many of your friends do you see or hear from at least once a month?<br>5. How many friends do you feel at ease with that you can talk about private matters?<br>6. How many friends do you feel close to such that you could call on them for help?	range: 0-30<br><br>0. none<br>1. one<br>2. two<br>3. three or four<br>4. five thru eigh<br>5. nine or more	
Italy	assist grandchildren/children (SP1)<br>bring food to children / grandchildren (if F) (SP2)<br>guests lunch / dinner (SP3)<br><br>number of weekly hours devoted to:<br>- find relatives (SP4)<br>- meet friends (SP5)<br>- playing cards (SP6)<br>- volunteering (SP7)<br>- cinema / theater / concerts / exhibitions (SP8)	1. yes, every day<br>2. yes, sometimes<br>3. yes, rarely<br>4. no<br><br>range: 0-168	

	- Mass / Church (SP9)		
Netherlands	Are you involved in one or more of these associations or organisations? (escocpyn)<br>- organization for the elderly (esocp01)<br>- trade union, employers organisation (esocp02)<br>- political party/organization (esocp03)<br>- church or religious organization (esocp04)<br>- neighbourhood association (esocp05)<br>- womens association (esocp06)<br>- organisation to assist elderly or disabled (esocp07)<br>- action group or association with social aim (esocp08)<br>- patients association (esocp09)<br>- choir, musical society, drama club (esocp10)<br>- hobby/social club (esocp11)<br>- sporting club (esocp12)<br>- other (esocp13)<br><br>Do you visit activities or meetings of:<br>-dito (esocp14-esocp26)<br><br>How often do you participate in activities or meetings of associations or organisations (esocp27)	-2. not available<br>0. not mentioned<br>1. mentioned<br><br>-3. not available, wrong skip<br>-2. not available<br>1. no<br>2. yes<br><br>-3. not available, wrong skip<br>-2. not available<br>1. almost never<br>2. a few times a year<br>3.<br>4.<br>5.<br>6. a few times a week<br>7. every day<br><br>-3. missing	

	How often did it occur in the last year that you helped someone with daily chores in and around the house? (eins_giv)	-1. no answer<br>1. never<br>2. rarely<br>3. sometimes<br>4. often	
Spain	PASE-items:<br>Caring for sick people frequency (pase_sick_fr)<br>Caring for children frequency (pase_child_fr)	0. never<br>1. <1/week<br>2. 1-2/week<br>3. 3-4/week<br>4. 5-7/week<br>9. no answer	
UK	Social activity score (soactiv). This score is derived from the social health questionnaire which includes the participation and frequency in:<br>- religious activities (sorelig, soreligf)<br>- positions of office (sopoff, sopofff)<br>- clubs (soclub, coclubf)<br>- courses (socours, socoursf)<br>- cultural visits (socult, socultf)<br>- indoor games (sogames, sogamesf)<br>- visiting friends (sovisit, sovisitf)<br>- pubs/social clubs (sopub, sopubf)<br>- individual hobbies (soindiv, soindivf)<br>- diy (sodiy, sodiyf)<br>- practical/craft hobbies (sopract, sopractf)<br>- gardening (sogard, sogardf)<br>- home computing (socomp, socompf)	Social activity score: range 0-100<br><br>Participation:<br>0. no<br>1. yes<br><br>Frequency:<br>3. weekly<br>4. monthly<br>5. less often	
	**Mastery**	Not available in 3 or more cohorts	Only available in NL and UK
Germany	Not available		
Italy	Not available		
Netherlands	Locus of control (Pearlin and Schooler, 1978) (emastery)<br>-Mastery item 01: control (emaster1)<br>-Mastery item 02: problems (emaster2)	-2. no valid data<br>range: 5 (low) - 25 (high)<br><br>1. strongly disagree	

	-Mastery item 03: changes (emaster3)<br>-Mastery item 04: helpless (emaster4)<br>-Mastery item 05: life (emaster5)<br>-Mastery item 06: everything (emaster6)<br>-Mastery item 07: future (emaster7)	2. disagree<br>3. no disagreement/ agreement<br>4. agree<br>5. strongly agree	
Spain	Not available		
UK	Derived from the social health questionnaire:<br>- control at home (sohmctrl)<br>- control at work (sowctrl)<br>- life beyond own control (sobeyond)<br>- more positives than negatives in next 5-10 yrs (sopositv)<br>- keeping healthy depends on what do (sohlthy)<br>- can reduce risk of heart attack (soriskmi)<br>- can reduce risk of cancer (soriskca)	1. strongly disagree<br>2. moderately disagree<br>3. slightly disagree<br>4. slightly agree<br>5. moderately agree<br>6. strongly agree<br><br>(It is advised to use the control at home variable only)	

RECEIVED CARE AND ASSISTENCE

	**Hospitalisation**	Note: reference period of hospitalisation differed across cohorts:<br>- past 12 months in Germany, Italy, Spain<br>- past 6 months in NL<br>- 12 months after baseline interview in UK<br>(hospital)	-1. missing<br>0. no<br>1. yes
Germany	During the past twelve months, have you stayed a night in a hospital? (IV1N181)<br>-how many nights in total? (IV1N183)	0. no<br>1. yes<br>range: 0-highest	0 → 0<br>1 → 1
Italy	hospitalization in the last year (HO1)	1. yes<br>2. no<br>9. don't know	1 → 1<br>2 → 0<br>9 → -1
Netherlands	Have been admitted to the hospital during the **past 6 months**? (ehosp)<br><br>Have you been admitted to a psychiatric hospital during the past 6 months? (epsyhosp)	-5. not available, interview terminated<br>-2. not available<br>1. no<br>2. yes<br>3. do not know	If yes on either somatic or psychiatric hospital → 1<br>If no on both somatic or psychiatric hospital → 0
Spain	Have you been hospitalized last year? (Hospitalisation)	1. yes<br>2. no<br>9. missing	2 → 0<br>1 → 1<br>9 → -1
UK	Hospital admission since baseline (HCS, 1999-2004)<br>- MSFU (2004/05) (fqhospad)<br>- COS (2007) (chospad)<br><br>Hospitalisation within 12 months **after** baseline interview (yes/no) (hcshosp)	0. no<br>1. yes<br><br><br>Composed by EPOSA<br>0. no<br>1. yes	 <br><br><br>0 → 0<br>1 → 1
	**Days of hospitalisation**	Note: reference period of hospitalisation is past 12 months in Germany, Italy, Spain<br>(hospdys)	-1. missing<br>Range: 0-365
Germany	During the past twelve months, have you stayed a night in a hospital? (IV1N181)	0. no<br>1. yes	If hospital=1 copy IV1N182

	-how many nights in total? (IV1N182)	range: 0-highest	
Italy	days hospitalization in the last year (HO2)	range: 0-highest	If hospital=1 copy HO2
Netherlands	Not available		missing
Spain	Hospitalisation days in one year (Hospitalisa_fr)	range: 0-highest<br>999. missing.	If hospital=1 copy Hospitalisa_fr
UK	Not available		missing
	**Contact health services**	Not available in 3 or more cohorts	Some info available in NL and Spain
	**Contact social services**	Not available in 3 or more cohorts	Only available in NL
	**Contact medical specialists**	Not available in 3 or more cohorts	Only available in NL, diagnosis-specific information available in Germany and Spain
	**Personal assistance**		Received domestic care (domestic)<br>-1. missing<br>0. no<br>1. yes, informal<br>2. yes, professional<br><br>Received personal care (personal)<br>-1. missing<br>0. no<br>1. yes, informal<br>2. yes, professional
Germany	If you need help or if you are being taken care of, who undertakes the care? (IV1N028, more than one answer allowed)<br><br><br><br><br><br><br><br>At your home, who performs tasks like cooking, ironing, cleaning, etc.?	1. no one, I do it myself<br>2. spouse<br>3. son/daughter<br>4. other relatives<br>5. social service<br>6. housekeeper/private nursing service<br>7. neighbour/friend<br>8. other person<br><br>1. I do it myself<br>2. spouse	Depends on data

	(IV1N032, more than one answer allowed)	3. relative<br>4. other person who lives in the apartment/house<br>5. paid worker<br>6. social service	
Italy	Help from a person:<br>*domestic.<br>- preparing food (IADL025)<br>- doing light work at home (IADL035)<br>- keeping the household accounts (IADL055)<br>*personal.<br>- moving in/out bed/chair (ADL014)<br>- walking into a room (ADL024)<br>- bathing or showering (ADL044)<br>- dressing or undressing (ADL054)<br>- eating (ADL064)<br>- using the toilet (ADL074)<br>- cutting toenails (ADL115)<br>- to take medication (IADL095)<br><br>Personal care: person who helps (ADL161)<br>Domestic care: person who helps (IADL111)	1. yes<br>2. no<br><br>1. son<br>2. daughter<br>3. wife<br>4. husband<br>5. daughter-in-law<br>6. other relatives<br>7. neighbour/friend<br>8. home care/social<br>9. salaried<br>10. other	Domestic care:<br>If "no difficulty" on all of the IADL-tasks → 0<br>If "yes" on any of the IADL-tasks and IADL111<=7 → 1<br>If "yes" on any of the IADL-tasks and IADL111>=8 → 2<br>If "yes" on any of the IADL-tasks and IADL111=10 → -1<br><br>Personal care:<br>If "no" on all of the ADL-tasks → 0<br>If "yes" on any of the ADL-tasks and ADL161<=7 → 1<br>If "yes" on any of the ADL-tasks and ADL161>=8 → 2<br>If "yes" on any of the ADL-tasks and ADL161=10 → -1
Netherlands	Are you getting help with:<br>-personal care (erhelpyn)<br>-domestic tasks (edhelpyn)<br>Care provided by:<br>- partner (erhelp01, edhelp01)<br>- resident child (erhelp02, edhelp02)	-5. not available, interview terminated<br>1. no<br>2. yes<br>3. don't know<br>4. refused	Domestic care:<br>If (edhelpyn=1) → 0<br>If (edhelpyn=2 and (edhelp01=2 or edhelp02=2 or edhelp03=2 or edhelp04=2 or edhelp05=2 or edhelp06=2 or edhelp07=2)) → 1<br>If (edhelpyn=2 and (edhelp08=2 or edhelp09=2 or

	- resident other (erhelp03, edhelp03)<br>- non-resident child (erhelp04, edhelp04)<br>- non-resident other relative (erhelp05, edhelp05)<br>- neighbours/acquaintances/friends (erhelp06, edhelp06)<br>- volunteers (erhelp07, edhelp07)<br>- district nurse (erhelp08, edhelp08)<br>- elderly/home/alpha help (erhelp09, edhelp09)<br>- private help (erhelp10, edhelp10)<br>- personnel home/hospital (erhelp11, edhelp11)<br>- other (erhelp12, edhelp12)	0. not mentioned<br>1. mentioned	edhelp10=2 or edhelp11=2)) → 2<br>If (edhelpyn=2 and edhelp12=2) → -1<br>If (edhelpyn=-5) → -1<br><br>Personal care:<br>If (erhelpyn=1) → 0<br>If (erhelpyn=2 and (erhelp01=2 or erhelp02=2 or erhelp03=2 or erhelp04=2 or erhelp05=2 or erhelp06=2 or erhelp07=2)) → 1<br>If (erhelpyn=2 and (erhelp08=2 or erhelp09=2 or erhelp10=2 or erhelp11=2)) → 2<br>If (erhelpyn=2 and erhelp12=2) → -1<br>If (erhelpyn=-5) → -1
Spain	Have you received help in the home? (help_home)<br><br><br><br>Was the help sent by<br>-social services (social_services)<br>-private service (private_services)<br><br>What type of help? (Help_1, Help_2)	1. yes<br>2. no<br>9. missing<br><br>1. yes<br>2. no<br>9. no answer<br><br>1. for care of the house<br>2. for personal hygiene<br>3. both<br>4. for an other cohabitant<br>9. no answer	Domestic care:<br>If (help_home=1 and (help_1=1 or help_2=1 or help_1=3 or help_2=3) and (social_services=1 or private_services=1)) → 2<br><br>If (help_home=1 and (help_1=1 or help_2=1 or help_1=3 or help_2=3) and (social_services=2 and private_services=2)) → 1<br><br>If (help_1~=1 and help_1~=3) and (help_2~=1 and help_2~=3) → 0<br>If (help_home=2) → 0<br><br>If (social_services=9 and private_services=9) → -1<br>If (help_1=9 and help_2=9) → -1<br>If (help_home=9) → -1<br><br>Personal care:<br>If (help_home=1 and (help_1=2 or help_2=2 or help_1=3 or help_2=3) and (social_services=1 or private_services=1)) → 2

			If (help_home=1 and (help_1=2 or help_2=2 or help_1=3 or help_2=3) and (social_services=2 and private_services=2)) → 1<br><br>If (help_1~=2 and help_1~=3) and (help_2~=2 and help_2~=3) → 0<br>If (help_home=2) → 0<br><br>If (social_services=9 and private_services=9) → -1<br>If (help_1=9 and help_2=9) → -1<br>If (help_home=9) → -1
UK			
	**Aids and adaptations**	Not available in 3 or more cohorts	Available in NL (detailed) and Spain (limited)
